# Modelling transmission of *Mycobacterium avium* subspecies *paratuberculosis* between Irish dairy cattle herds

**DOI:** 10.1186/s13567-022-01066-5

**Published:** 2022-06-22

**Authors:** Floor Biemans, Jamie Tratalos, Sandie Arnoux, George Ramsbottom, Simon J. More, Pauline Ezanno

**Affiliations:** 1 Centre for Veterinary Epidemiology and Risk Analysis, UCD School of Veterinary Medicine, University College Dublin, Belfield, Dublin, D04 W6F6 Ireland; 2 INRAE, Oniris, BIOEPAR, 44300 Nantes, France; 3 Teagasc, Oak Park, Carlow, Ireland

**Keywords:** Johne’s disease, stochastic model, dairy cows, infectious disease, data driven

## Abstract

Bovine paratuberculosis is an endemic disease caused by *Mycobacterium avium* subspecies *paratuberculosis* (*Map*). *Map* is mainly transmitted between herds through movement of infected but undetected animals. Our objective was to investigate the effect of observed herd characteristics on *Map* spread on a national scale in Ireland. Herd characteristics included herd size, number of breeding bulls introduced, number of animals purchased and sold, and number of herds the focal herd purchases from and sells to. We used these characteristics to classify herds in accordance with their probability of becoming infected and of spreading infection to other herds. A stochastic individual-based model was used to represent herd demography and *Map* infection dynamics of each dairy cattle herd in Ireland. Data on herd size and composition, as well as birth, death, and culling events were used to characterize herd demography. Herds were connected with each other through observed animal trade movements. Data consisted of 13 353 herds, with 4 494 768 dairy female animals, and 72 991 breeding bulls. We showed that the probability of an infected animal being introduced into the herd increases both with an increasing number of animals that enter a herd via trade and number of herds from which animals are sourced. Herds that both buy and sell a lot of animals pose the highest infection risk to other herds and could therefore play an important role in *Map* spread between herds.

**Supplementary Information:**

The online version contains supplementary material available at 10.1186/s13567-022-01066-5.

## Introduction

Bovine paratuberculosis, or Johne’s disease, is a disease caused by *Mycobacterium avium* subspecies *paratuberculosis* (*Map*). Johne’s disease is endemic in the dairy sector worldwide. It has a large economic impact due to milk losses, early culling and increased mortality [1]. Susceptibility reduces with age, and animals are usually infected in the 1^st^ year of life [2]. Clinical signs, including weight loss, decreased milk production, and diarrhoea, do not usually appear before first calving and are sometimes never observed [3]. However, shedding starts before animals show clinical signs [4]. Tests to identify infected animals include milk or serum ELISA, PCR, and faecal culture. However, diagnosing infected animals is difficult because of the low sensitivity of these tests, ranging from 15–71% depending on the stage of infection [5, 6]. Thus, infectious animals can remain undetected in a herd. *Map* is mainly transmitted between herds through movement of these infected but undetected animals [7]. Understanding the effect of animal movements on the spread of *Map* between herds and its subsequent persistence within infected herds can be very informative for control purposes. Once *Map* is introduced into a herd, model results suggest that a herd can remain infected for over 10 years [8, 9]. Indeed, the probability of persistence 15 years after introduction has been estimated to be up to 42.7% following the introduction of a single infected animal into a typical spring calving Irish dairy herd [10].

In this study we aim to investigate *Map* spread between Irish dairy herds. In Ireland, the dairy industry is pasture-based to optimize the use of grass as the primary feed source for lactating cattle [11]. For more than 90% of dairy herds, cows are housed during the winter, and most calves are born in spring [11, 12]. Herds have the opportunity to join the voluntary Irish Johne’s Control Programme (IJCP), which has four objectives: (1) Enhance the ability of farmers to keep their herds free of *Map*, (2) Reduce the level of infection in infected herds, (3) Provide additional assurance to the marketplace, and (4) Improve calf health and farm biosecurity [13, 14]. At the end of 2020, 11% of the dairy herds in Ireland, representing 18% of the dairy cows, were registered in the IJCP [13]. Herd prevalence was estimated to be 20.6% in 2005 [15] and 28% in 2013–2014 [16].

Although trade movements are well described in Ireland [17], the contribution and intensity of trade movements between herds on *Map* spread at the national scale in Ireland are still barely understood. Epidemiological modelling is relevant to answering this question, as it enables us to integrate both the within-herd population and infection dynamics, and to link herds through trade movements [18]. So far, only a few such models have been developed to predict *Map* spread between cattle herds at a large scale, including a model adapted to the dairy farming system of western France [19, 20], a model for Northern Italy [21], and a model for Slovenia [22, 23]. The French model links stochastic compartmental within-herd models to each other using cattle trade data. Recently, this model has been transformed into an individual-based model to better account for animal characteristics and to facilitate assessment of control measures [24, 25]. The flexibility of this model makes it a good candidate to be adapted to represent *Map* spread in the predominantly seasonal Irish dairy farming system [26].

We used an adapted version of the French transmission model to simulate *Map* spread. Our objective was to investigate the effect of herd characteristics on *Map* spread in dairy herds in Ireland. Herd characteristics that could be calculated from the movement data included herd size, number of male animals introduced for breeding, number of animals purchased and sold, and number of herds that the focal herd purchases from and sells to. We used these characteristics to classify herds in accordance with their probability of becoming infected and of spreading infection to other herds.

## Materials and methods

A stochastic individual-based model was used to represent herd demography and *Map* infection dynamics of each dairy cattle herd in Ireland [10, 24]. Data on herd size and composition, as well as birth, death, and culling events were used to characterize herd demography. Herds were connected with each other through observed animal trade movements [17]. A comprehensive dataset on cattle trade movements at a national scale in Ireland was used, comprising information for each moved animal (e.g., age at move, sex, breed, date of move, reason for the move, and source and destination herd). Details on the datasets used, and on the processes occurring at within- and between-herd scale are presented hereafter.

### Data

Data were extracted from the Animal Identification and Movement system (AIM) of the Irish Government’s Department of Agriculture, Food and the Marine. The AIM database comprises records on bovine births, movements and movement types [17, 27]. Movement data and demographic data (e.g., animal ID, sex, breed, date of birth) were extracted for the 10-year period 1^st^ January 2009–31^st^ December 2018.

Herds included in our dataset, also referred to as the studied metapopulation, were dairy herds classified as such using a similar methodology to Brock et al. [28]. In general, European dairy herds are organized into groups of animals that are a similar age. Each group has its own housing and management that influence how, and with which other animals, they can interact [29]. The model was developed to simulate this contact structure for typical European dairy herds [9, 24, 30]. Therefore, only dairy herds were included.

**Figure 1:**
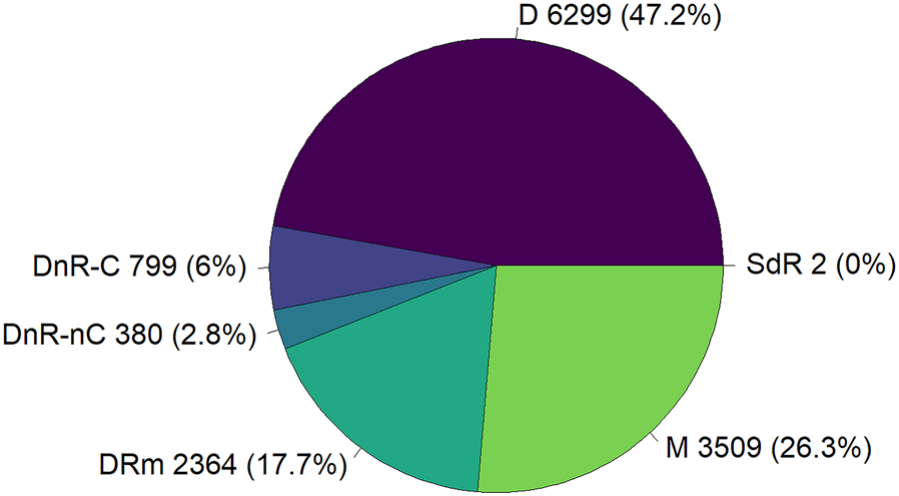
**Distribution of herds included in the metapopulation per herd type**. Herd types (Table 1) include typical dairy (D), dairy no rearing—contract (DnR-C), dairy no rearing—no contract (DnR-nC), dairy also rearing male calves (DRm), mixed (M), and herds that rear dairy females (SdR). For each herd, its herd type was determined based on data from 2017.

Only herds with more than half of their animals of dairy breed (Additional file 1) were selected. Furthermore, more than 30% of the animals had to be female. With these two criteria, we were able to separate mixed herds (with female and male animals of dairy and beef breed) from store dairy male herds (high proportion of males that are of dairy breed [28]) and from store beef mixed herds (high proportion of beef animals that could be either males or females [28]). Next, herds were selected based on completeness of movement data (data were considered complete when data were available for a herd for each year of the study period). To simplify the simulations and analyses, we chose to exclude herds that closed or started during the study period (2127 herds). A herd was considered to be closed when, over the entire study period, animals only entered the herd via birth and exited the herd via death or slaughter. Because these herds do not trade with other herds, they do not contribute to *Map* spread and were excluded from the dataset. Only female animals were modelled, with the exception of the purchase of some male animals for breeding (see below). Hence, herds that did not exchange female animals with other herds were also excluded. Finally, herds were selected on the basis of size: herds with 15 animals or less, or five adults or less, were considered to be non-commercial dairy herds and were excluded. A total of 13 353 herds, with 4 494 768 dairy female animals, were included in the metapopulation. The retained herds belonged to one of six possible herd types (defined in Table 1), as described in Brock et al. [28]. Figure 1 presents the distribution of herds included in the metapopulation per herd type. Some farms within the metapopulation conducted both dairy and fattening activities, however, for the purpose of modelling, it was assumed that the fattening activities took place on a different part of the farm with negligible contact between the dairy and fattening herds, and thus the fattening activities were not represented in the model.

**Table 1:** **List of herd types**

Herd type	Abbreviation	Description
Typical dairy	D	Female dairy calves are reared to become replacement heifers and most male calves are sold at an early age. There are almost no males between the age of 1 and 2 years
Dairy no rearing—contract	DnR-C	Sell most of their calves, with female dairy calves being moved to external rearing herds. Female calves return to their birth herd as pregnant heifers
Dairy no rearing—no contract	DnR-nC	Cows are bred to beef bulls and most of their calves are sold. Replacement animals are bought from herds with a surplus of cows or pregnant heifers
Dairy rearing males	DRm	Female dairy calves are reared to become replacement heifers and males are kept. There is a high proportion of male animals between the age of 1 and 2 years
Mixed	M	Have both milk and beef production activities. Have pure-bred dairy females and cross-bred dairy and beef animals
Store dairy rearing	SdR	Female dairy calves are reared and inseminated before returning to their birth herd. Animals are usually of young age

A full description of these herd types can be found in [28].

In Ireland, the use of natural service bulls following rounds of artificial insemination is common practice [31]. To account for the risk of introducing *Map* into a herd via the purchase of bulls for breeding purposes, some males were also included in the dataset. Including bulls was only considered for herds that purchased dairy or beef bulls (83% of the herds). To differentiate bulls introduced for fattening purposes from bulls introduced for breeding purposes, only bulls that met the following criteria were included: bulls had to be pure bred dairy or beef, they had to enter the herd via trade, and beef bulls had to come from a non-dairy breeder herd. Bulls had to be at least 1 year of age upon entering a herd, and they had to remain in a herd for at least 5 months (one breeding season). The maximum length of stay in a herd was 4 years for beef bulls and 2 years for dairy bulls. It was assumed that dairy bulls were only used for breeding for a maximum of 2 years because after that these animals had the potential to mate with their daughters (who first calve at approximately 2 years of age). The number of bulls introduced per year was calculated based on the assumption that about half the cows would be serviced by a bull, with 20 cows per bull. The other half of the cows will be artificially inseminated. The number of cows present in a herd was determined on the first of May of a given year. Based on this calculation, if more bulls were needed than present in the trade data, dairy bulls that were born into a herd and kept until breeding age were selected. A total of 72 991 bulls were selected for 11 121 herds of the metapopulation and added to the dataset.

### Within-herd *Map* transmission model

The within-herd model is adapted from Camanes et al. [24] and Biemans et al. [10]. All parameters used and model equations related to disease transmission are described in detail in the supplementary material of Biemans et al. which is open access [10]. Please refer to that study for a detailed overview. Briefly, it is a stochastic individual-based model with a discrete time step of 1 week. A time step of 1 week was chosen because it makes it possible to accurately represent the susceptible decay and the seasonal herd management of Irish dairy herds while reducing computation time compared to a time step of, e.g., 1 day. It accounts for herd structure and infection dynamics (Additional file 2). Animals belong to one of six age groups: newborn calves, unweaned calves, weaned calves, young heifers, bred heifers, and cows. Depending on the age group that animals are in, they move to the next age group either because they reach a defined age or at a defined time in the year (Additional file 2). Animals also belong to one of six health states: susceptible (S), resistant (R), transiently infectious (I_T_), latently infected (I_L_), moderately infectious (I_M_), and highly infectious and with the possibility to also be clinically affected (I_H_). Animals are assumed to be most susceptible at birth, with susceptibility decreasing exponentially with age [2]. Infectious animals (I_L_, I_M_, I_H_) shed *Map* in their faeces and, after calving, in their colostrum and milk. The quantity shed depends on the health state and is heterogeneous between animals of the same state [4, 32]. Animals can get infected via the following transmission routes: in utero including transmission during parturition, via ingestion of contaminated colostrum or milk (directly or indirectly with faeces), via contact with the local environment or the general indoor environment contaminated with faeces. Transmission via the local environment is defined as the risk posed by other animals held in the same place but not necessarily at the same time. The local environment can be indoor or on pasture. Transmission via the general indoor environment is defined as the risk posed by other animals held indoors but not necessarily at the same place or time. All infectious animals that reside indoors contribute to the contamination of the general indoor environment. Within the indoor environment, 40% of the *Map* load present was removed per week, representing the effect of removing manure [9]. Similarly, on pasture, a 7.1% reduction in *Map* load occurred weekly [33]. Additionally, when there are no animals present in one of the indoor environments, they are cleaned more thoroughly, removing an extra 16.7% of the *Map* load [10, 30]. Details of all model parameters are in Additional file 1.

To represent Irish dairy farm management, which is highly seasonal [26], animals are divided into two clusters depending on the season in which they are born in order to prevent mixing of yearlings. The spring cluster consists of animals born in the first half of the year, while the autumn cluster consists of animals born in the second half of the year. The cluster, animal age, and the time of the year determine the age group and environment of an animal, e.g., spring-born unweaned calves are kept indoors up to week 14 of the year and on pasture from week 14 onwards, whereas autumn-born unweaned calves are always kept indoors. For both clusters, details of the transitions between age classes and environments in which animals are kept are in Additional file 2.

For each herd in the metapopulation, herd parameters for size and exit rates were calculated from the data. Herd size was calibrated on the 1^st^ of January 2009. For each age class, seasonal exit rates (i.e., exit rates over a 3-month period; January–March, April–June, July–September, October–December) were defined because it resulted in the best agreement between observed and modelled herd size compared to the use of yearly or monthly exit rates (Additional file 3). A newborn calf was added to the herd whenever a birth was observed in the data.

### Between-herd *Map* transmission model

The between-herd model connects all the within-herd dynamics through animal movements. Movements from and to a herd in the metapopulation are modelled as observed in the data. When an animal was moved via a market, the movement was represented as if it occurred directly from the source herd to the destination herd. The movement dataset comprises 2 304 149 animal movements of which 17% were between herds in the metapopulation, 20% were from a herd outside of the metapopulation to a herd in the metapopulation, and 63% were from a herd in the metapopulation to a herd outside of the metapopulation.

The movement data define the date that a movement occurs, the age of the animal moved, and the source and destination herd. The animal to be moved is randomly selected from the relevant age group in the source herd, and thus can be of any health state. When there is, due to chance, no animal present in the correct age group, an animal of the closest age group is selected. If an animal is coming from a herd outside of the metapopulation, its health state is drawn from a distribution that corresponds to the proportion of animals in each health state in the same age group within the entire metapopulation. Thus, it is assumed that the average risk of introducing an infected animal is the same within and from outside of the metapopulation.

### Herd characteristics

Seven herd characteristics are calculated from the movement data for every year: in-degree, out-degree, in-strength, out-strength, polarity, herd size, and number of male animals introduced. The in-degree measures the number of herds that the herd of interest receives animals from. The out-degree measures the number of herds that the herd of interest sends animals to. The in-strength measures the number of animals purchased by the herd of interest (incoming movements). The out-strength measures the number of animals sold by the herd of interest (outgoing movements). Polarity is defined as $Polarity=\frac{\# incoming\, animals-\# outgoing\, animals}{\# incoming\, animals+\# outgoing\, animals}$. Polarity takes values between −1 and 1 and represents the trading behaviour of a herd, where herds with a polarity between −1 and −0.25 can be considered as predominantly selling herds, between −0.25 and 0.25 as neither predominantly buying or selling herds, and between 0.25 and 1 as predominantly buying herds [34]. The herd size was calculated as the total number of animals present in a herd on the first of January. These herd characteristics are calculated for every year and then averaged over the 10-year period. The annual strength and degree can vary over the years. The median values for the difference between the lowest and the highest value were 2.0 for in-degree and out-degree, 9.0 for in-strength, and 18.0 for out-strength (Additional file 4).

### Simulation settings

Which herds were chosen to be infected at the start of the simulations had an effect on model predictions (Additional file 5). Therefore, we first assessed which herds were the most likely to be infected 10 years after *Map* introduction in the metapopulation when they were not the ones initially infected. On this basis, for 1000 replicates, 25% of the herds were randomly chosen to be initially infected irrespective of their herd characteristics. The within-herd prevalence is low in the majority of herds based on earlier analyses of field data [15, 35]. Therefore, the within-herd prevalence in the herds that are initially infected was drawn from a Gaussian distribution N(−0.42,0.12), keeping only values below 0.7 because higher values are rarely observed in the field (Additional file 6). The probability of a herd being infected after 10 years of simulating *Map* transmission was calculated for each herd given that it was not initially infected. The 30% most likely herds to be infected were chosen as candidate herds to be initially seeded with *Map* in the subsequent simulations.

Second, for 300 replicates, amongst the candidate herds that were most likely to be infected, 25% of the total number of herds were chosen to be initially infected. Their initial within-herd prevalence was drawn from the aforementioned distribution. Assuming a herd prevalence of 25% in 2009 was in agreement with field observations, the prevalence being estimated at 20.6% in 2005 [15], while it has been estimated at 28% in 2013–2014 [16]. *Map* transmission was simulated for 10 years, matching the temporal extent of the movement data.

### Model outputs

Firstly, we analysed over time the proportion of herds infected, hereafter called “herd prevalence”, and the proportion of infected animals among > 2-year-old animals present in each infected herd the first of January each year, hereafter called “within-herd prevalence”. Secondly, we investigated the correlation between observed herd characteristics to provide context when interpreting the results of the simulations. Thirdly, we calculated for each herd the probability of becoming infected and the probability of escaping infection. We related these probabilities to the in- and out-degrees, in- and out-strengths, herd size, and number of male animals introduced for breeding. The probability of a herd becoming infected was calculated with as denominator the number of replicates in which a herd was not initially infected and as numerator the subset of these replicates in which at least one infected animal entered the herd during the 10-year simulation period. The probability of a herd escaping infection was calculated using the same denominator (the number of replicates in which a herd was not initially infected) and as numerator the subset of these replicates in which no infected animal entered the herd within the 10-year simulation period. A generalized linear model with a logit link was used to connect (the logarithm of) each herd characteristic to the probability of a herd being infected, model fit was assessed by Akaike information criterion (AIC). Fourthly, we investigated the infection risk posed by each selling herd type. We determined the number of buying herds infected following outward movements of infected animals from each selling herd, and summarised as the average number of herds infected by each selling herd type. A buying herd was considered to be infected by the selling herd if at least one infected animal was introduced and up to that moment no infected animals had been present in the herd. Lastly, we determined the number of unique infection sources per herd. A herd is an infection source if it introduces an infected animal in the herd of interest in at least one replicate.

Results were analysed and visualized in R [36] using the dplyr, tidyr, ggplot2, viridis, ggridges, PerformanceAnalytics, multcomp and Reshape2 packages [37–44].

## Results

### Prevalence

**Figure 2:**
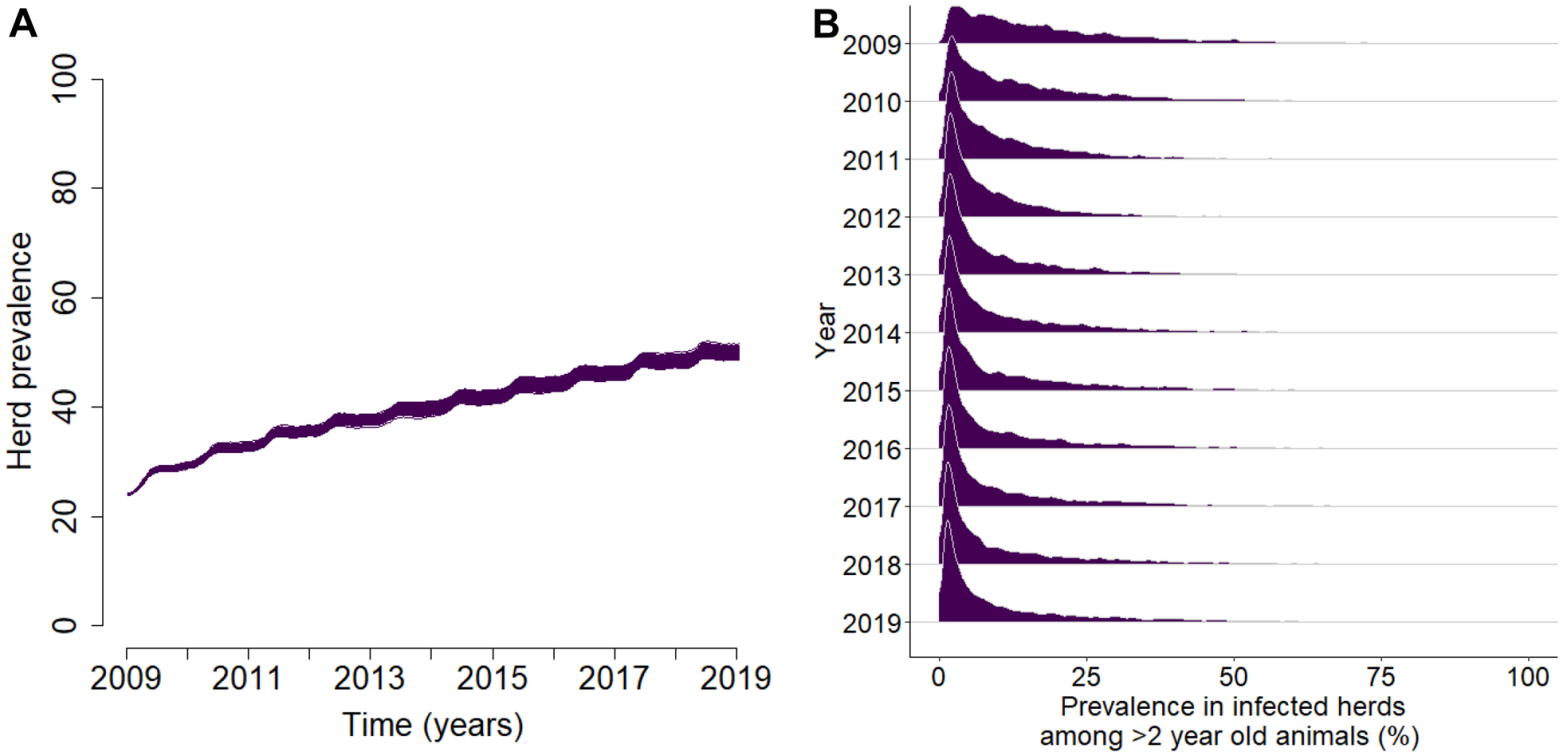
**Herd prevalence and within herd prevalence over time.** **A** Herd prevalence for all replicates. **B** Distribution of within-herd prevalence within infected herds, based on infection prevalence among > 2-year-old animals on the 1^st^ of January of the respective years.

For all replicates, herd prevalence increased over time (Figure 2A). After 10 years of simulation, herd prevalence was 49.9% on average. The average within-herd prevalence among > 2-year-old animals for all herds (including uninfected herds) was 4.1% at the start of the simulations in January 2009. It increased to 5.6% at the end of the simulations in December of 2018. The average within-herd prevalence among > 2-year-old animals within infected herds (Figure 2B) was 17.8% in 2009, decreasing to 8.5% in 2012 then increasing to 12.7% at the end of 2018. This initial decrease was expected as only few infected animals are present in newly-infected herds, which by calculation induces a decrease in the average within-herd prevalence.

### Herd characteristics

Table 2 presents the mean value of each herd characteristic per herd type. Herds classified as SdR (herds that rear dairy females) have the highest values for in-strength and out-strength but the number of SdR herds is small (*n* = 2). On average, herds classified as DnR-C (dairy herds which contract out calf rearing to other herds) have a higher out-degree and out-strength compared to the other herd types. Furthermore, herds classified as DnR-C or DnR-nC (non-rearing dairy herds which do not use contract rearing) have a higher in-strength compared to the other herd types.

**Table 2:** **Mean value of a herd characteristic per herd type**

Herd type	In-degree	Out-degree	In-strength	Out-strength	Herd size	Number of males purchased
D	1.07 *(0.30–1.20)*	1.92 (1.00–2.20)	4.99 (0.45–4.65)	15.79 (5.90–19.40)	125.6 (72.0–153.9)	0.52 (0.10–0.70)
DnR-C	1.18 (0.70–1.40)	2.37 (1.10–2.80)	22.29 (8.55–27.6)	43.05 (19.30–54.00)	167.5 (99.5–204.7)	0.87 (0.30–1.20)
DnR-nC	2.90 (0.90–2.70)	1.59 (1.00–1.80)	15.08 (3.40–14.43)	15.71 (4.28–15.45)	77.4 (41.2–88.8)	0.40 (0.10–0.50)
DRm	1.41 (0.30–1.30)	1.44 (0.50–1.60)	5.58 (0.60–4.70)	9.75 (2.30–12.40)	118.9 (73.1–146.7)	0.70 (0.20–1.00)
M	1.40 (0.50–1.60)	1.03 (0.50–1.30)	4.78 (1.00–5.80)	5.82 (1.80–7.70)	79.3 (47.1–100.0)	0.49 (0.10–0.60)
SdR	2.45 (1.58–3.33)	1.35 (1.18–1.53)	77.20 (53.70–100.70)	82.60 (52.45–112.75)	62.3 (58.6–66.0)	0.15 (0.08–0.23)
Overall	1.27 (0.40–1.40)	1.62 (0.80–1.90)	6.37 (0.70–6.20)	13.74 (3.70–16.30)	113.3 (62.9–139.3)	0.56 (0.10–0.80)

The 25% and 75% quantile values are in parentheses. Herd types are typical dairy (D), dairy no rearing—contract (DnR-C), dairy no rearing—no contract (DnR-nC), dairy also rearing male calves (DRm), mixed (M), and herds that rear dairy females (SdR) (defined in Table 1).

**Figure 3:**
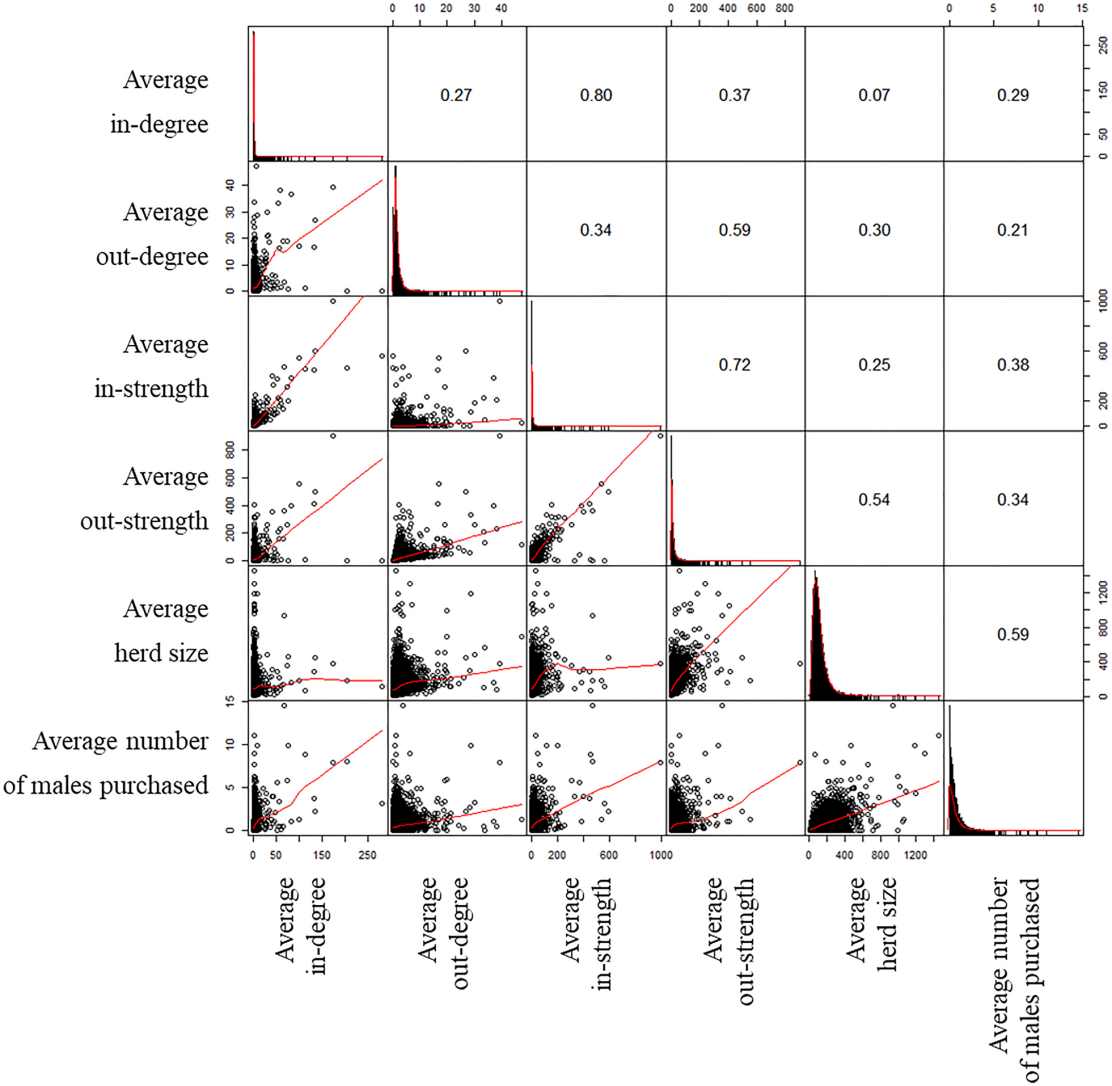
**Visualization of the correlation matrix between the six herd characteristics as observed in the dataset for all 13 353 herds**. On the diagonal are histograms of the values of the herds characteristics, to the right of the diagonal are the Pearson correlation coefficients, and to the left are bivariate scatterplots with a fitted line (red).

Figure 3 presents the correlation between the six herd characteristics for all 13 353 herds. In-degree and in-strength have a strong positive correlation (0.80), as have in-strength and out-strength (0.72). For the other characteristics, the correlation is moderate (0.36–0.67) to weak (< 0.36).

### Probability of becoming infected and probability of escaping infection

**Figure 4:**
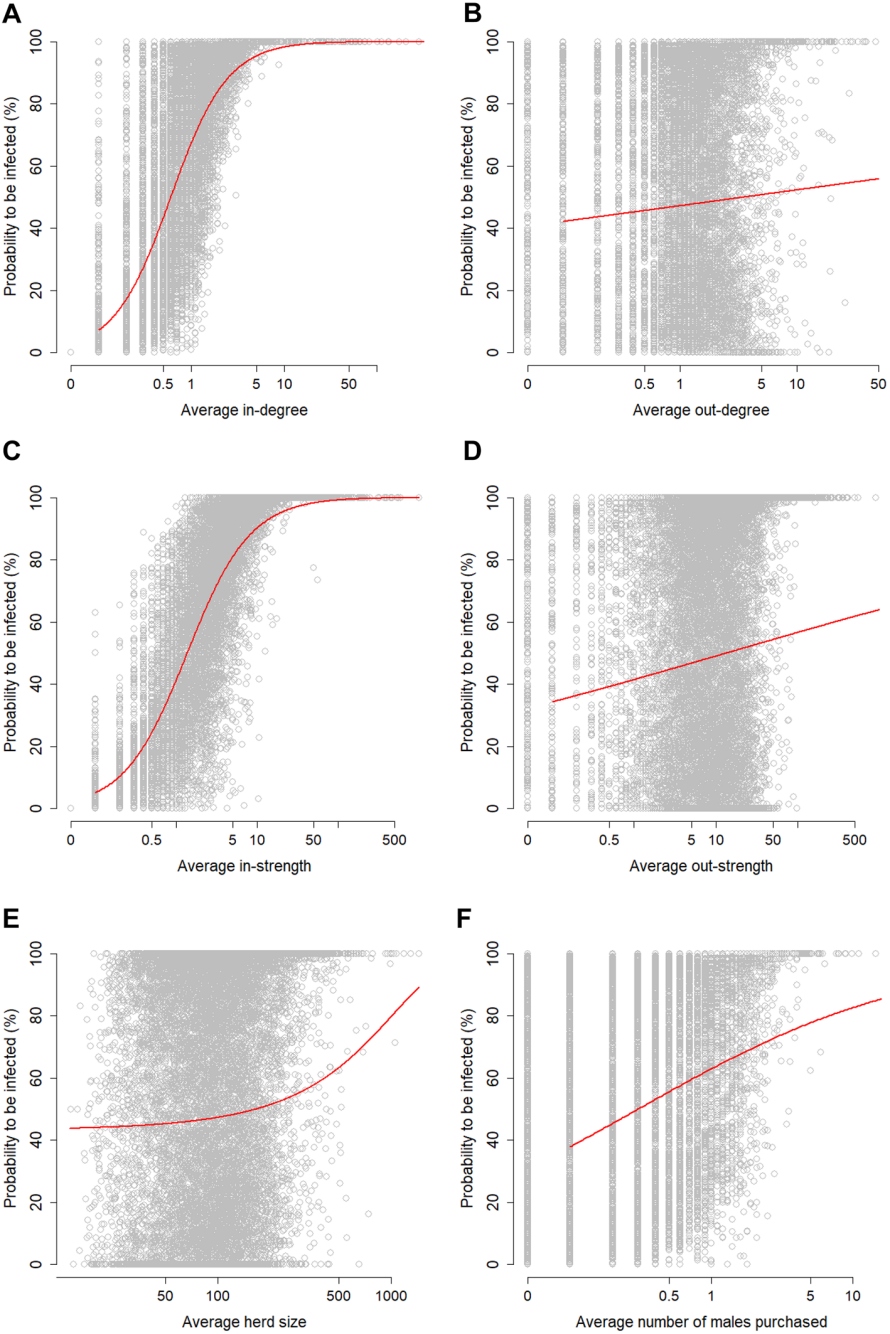
**Probability of a herd becoming infected versus six herd characteristics**. Each point represents a single herd. Red lines correspond to the prediction of the generalized linear model with a logit link and the logarithm of the herd characteristic (in-degree (**A**), out-degree (**B**), in-strength (**C**), out-strength (**D**), number of males purchased (**F**)) or the herd characteristic (herd size (**E**)) as explanatory variable. For the definition of the probability of a herd becoming infected see text.

Figure 4 presents the probability of a herd becoming infected versus six herd characteristics. For all of these herd characteristics, the probability to becoming infected increased with higher values for the characteristics. For all models, the explanatory variables were significant, but the model with the logarithm of in-strength as the explanatory variable had the best fit (lowest AIC). For 957 herds, the probability to becoming infected was zero. However, this did not necessarily mean that they did not purchase any animals, as 177 of these herds had an average in-degree (mean = 0.15) and in-strength (mean = 0.36) higher than 0. For 2390 herds, the probability to becoming infected was 100%. However, this did not necessarily mean that they purchased animals from many other herds, as 481 of these herds (20.1%) had an average in-degree lower than 1 (mean = 0.69). Herds with an average in-degree higher than 3, herds with an in-strength higher than 8 and herds that purchased more than three breeding bulls per year all had a probability exceeding 95% of becoming infected at least once during the 10 years of simulation.

**Figure 5:**
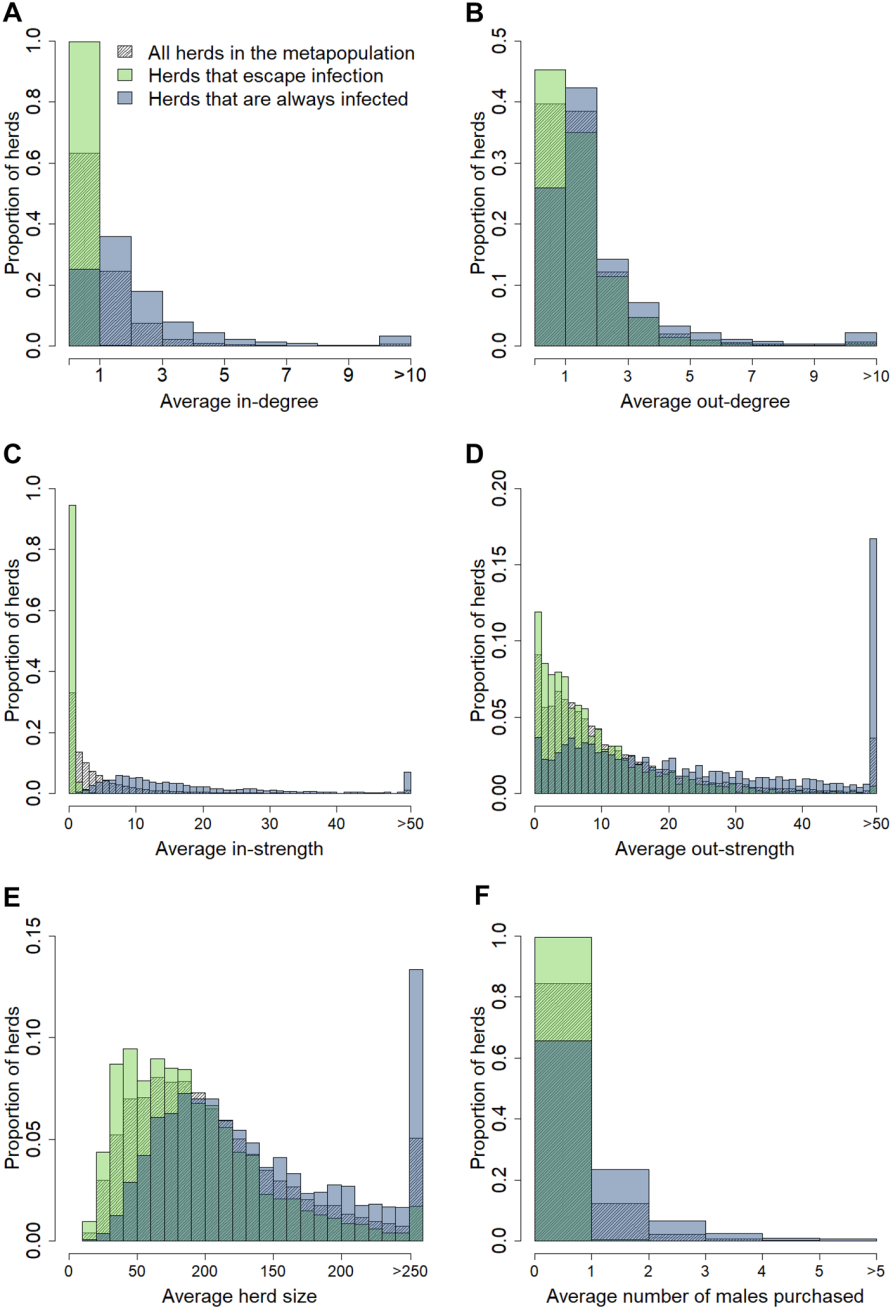
**Distribution of herd characteristics among all herds, those escaping infection, and those always infected.** Herd characteristics of all herds in the metapopulation (n = 13 353, striped), herds that have a probability > 90% of escaping infection (1629 herds, green), and herds that consistently became infected during the 10-years simulation period (3862 herds, blue). Herd characteristics considered: average in-degree (**A**), average out-degree (**B**), average in-strength (**C**), average out-strength (**D**), average herd size (**E**), and average number of males purchased (**F**). For the definition of the probability of a herd escaping infection or becoming infected see text.

Of the 13 353 herds in the metapopulation, 1969 herds escaped infection in more than 90% of the replicates. Figure 5 presents the distribution of herd characteristics among these 1969 herds compared to all herds in the metapopulation, and to herds that became infected during the 10-year simulation period in every replicate. For every herd characteristic, the average value was the lowest for herds that escaped infection and the highest for herds that always became infected. This difference was most pronounced for the average in-strength, with a median value of 0.1 for herds that escaped infection and 14.0 for herds that always became infected. Of the herds that escaped infection, 57.2% never purchased a male animal, while only 4.4% of the herds that were always infected never purchased a male animal. For 99.5% of the herds that escaped infection, the average in-strength was lower than 4, while for 96.2% of the herds that always became infected the average in-strength was 4 or more.

### The role of the different herd types on *Map* spread between herds

**Figure 6:**
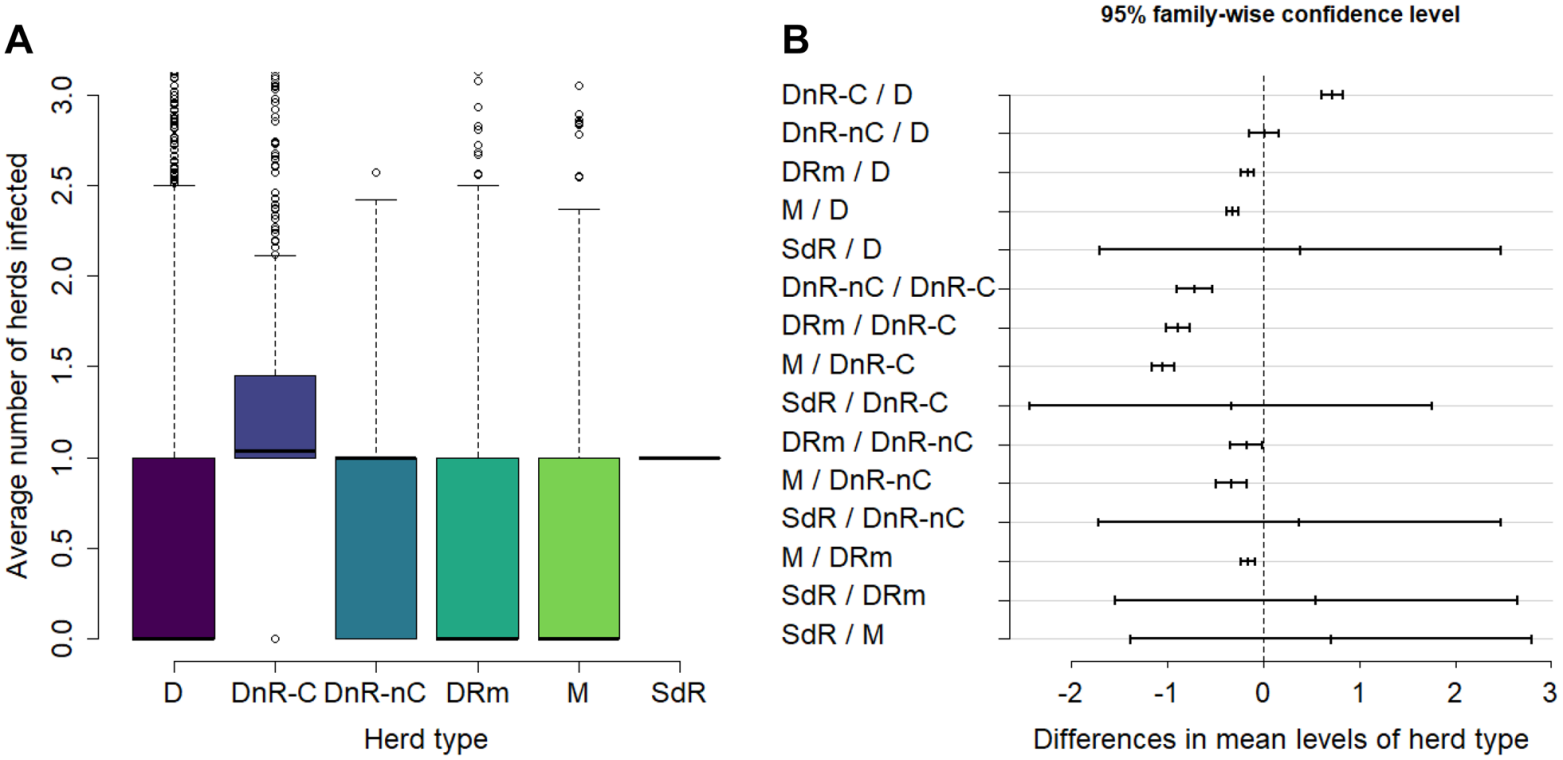
**Distribution of the infection risk posed by each selling herd type.** **A** Infection risk posed per herd type. Each box contains values between the first and the third quartiles, the horizontal line corresponding to the median. Vertical lines outside the boxes extend to 10^th^ and 90^th^ percentiles. **B** Tukey test-95% family-wise confidence level for each combination of herd types. The figure presents the value of the difference between the means and their respective 95% CI. The vertical dashed line indicates the point where the difference in means is zero. Herd types are described in Table 1. For the definition of infection risk posed see text.

Figure 6 presents the distribution of the infection risk posed by each selling herd type. Herds classified as DnR-C infected significantly more dairy herds on average compared to all other herd types. Herds classified as DnR-nC and herds classified as typical dairy (D) infected significantly more herds than mixed herds (M) and dairy herds that also rear male calves (DRm). DRm herds infected significantly more dairy herds than mixed herds. Thus, for the mean average number of dairy herds infected per herd type: DnR-C > DnR-nC and D > DRm > M. The percentage of herds per type that did not infect any other dairy herds was 23.0% for DnR-C, 48.2% for DnR-nC herds, 54.1% for D, 65.6% for DRm, and 74.1% for M. The DnR-C herds that infected other dairy herds infected 1.7 dairy herds on average, with a maximum of 29.9. For all herd types except SdR, herds that infected other dairy herds infected between 1.1 and 1.3 herds on average. The mean of herds classified as SdR (*n* = 2) was not significantly different from any other herd type.

**Figure 7:**
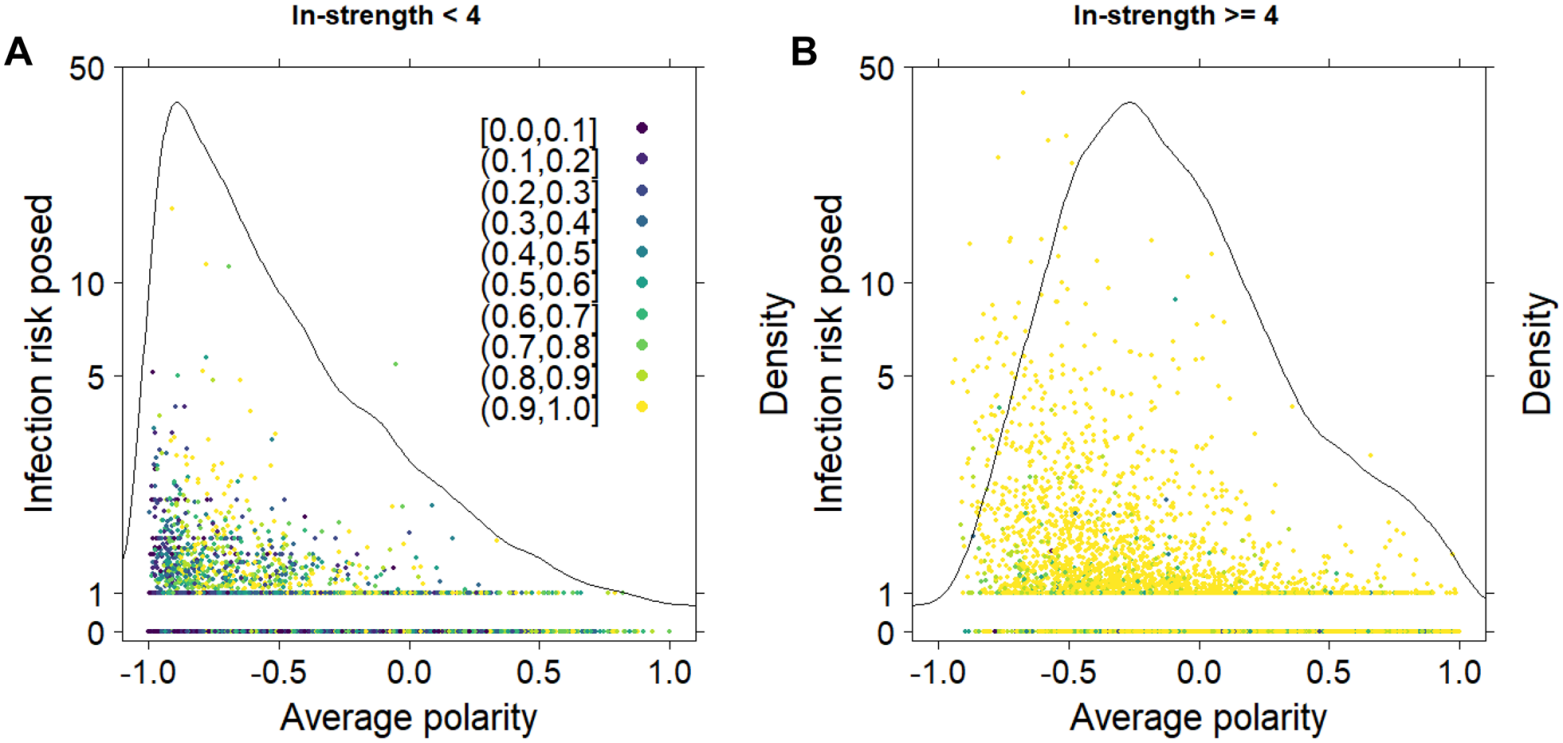
**Infection risk versus average polarity.** **A** Herds with an average in-strength of less than 4 (*n* = 8478); **B** Herds with an average in-strength of 4 or more (*n* = 4875). Polarity is an indicator of the trading behaviour of a herd, where herds with a polarity between −1 and −0.25 can be considered as predominantly selling herds, between −0.25 and 0.25 as neither predominantly buying or selling herds, and between 0.25 and 1 as predominantly buying herds. Points are coloured according to the probability of a herd being infected. Lines show the density curve. For the definition of infection risk posed see text.

Figure 7 presents the average number of susceptible herds in which the herd of interest (the selling herd) introduced an infected animal versus the average polarity of the herd of interest. Herds with an average in-strength of less than 4 are presented separately from herds with an average in-strength of 4 or more, based on the results presented in Figure 5C. Herds could have a very different in-strength while having the same polarity, e.g., a herd that sells one animal and buys one animal has a polarity of 0, while a herd that sells 50 animals and buys 50 animals also has a polarity of 0. However, the risk of becoming infected and spreading infection is not the same. The mean polarity of herds with an in-strength of less than 4 was −0.49, and for herds with an in-strength of 4 or more it was −0.09. The mean probability of becoming infected during the 10-year simulation period was 40.0% for herds with an in-strength of less than 4, and 93.2% for herds with an in-strength of 4 or more. The mean number of susceptible herds into which an infected animal was introduced was 0.34 for selling herds with an in-strength of less than 4 and 70% of these selling herds did not infect any other herd. For selling herds with an in-strength of 4 or more this number was 0.89, and 41% of these selling herds did not infect any other herds.

**Figure 8:**
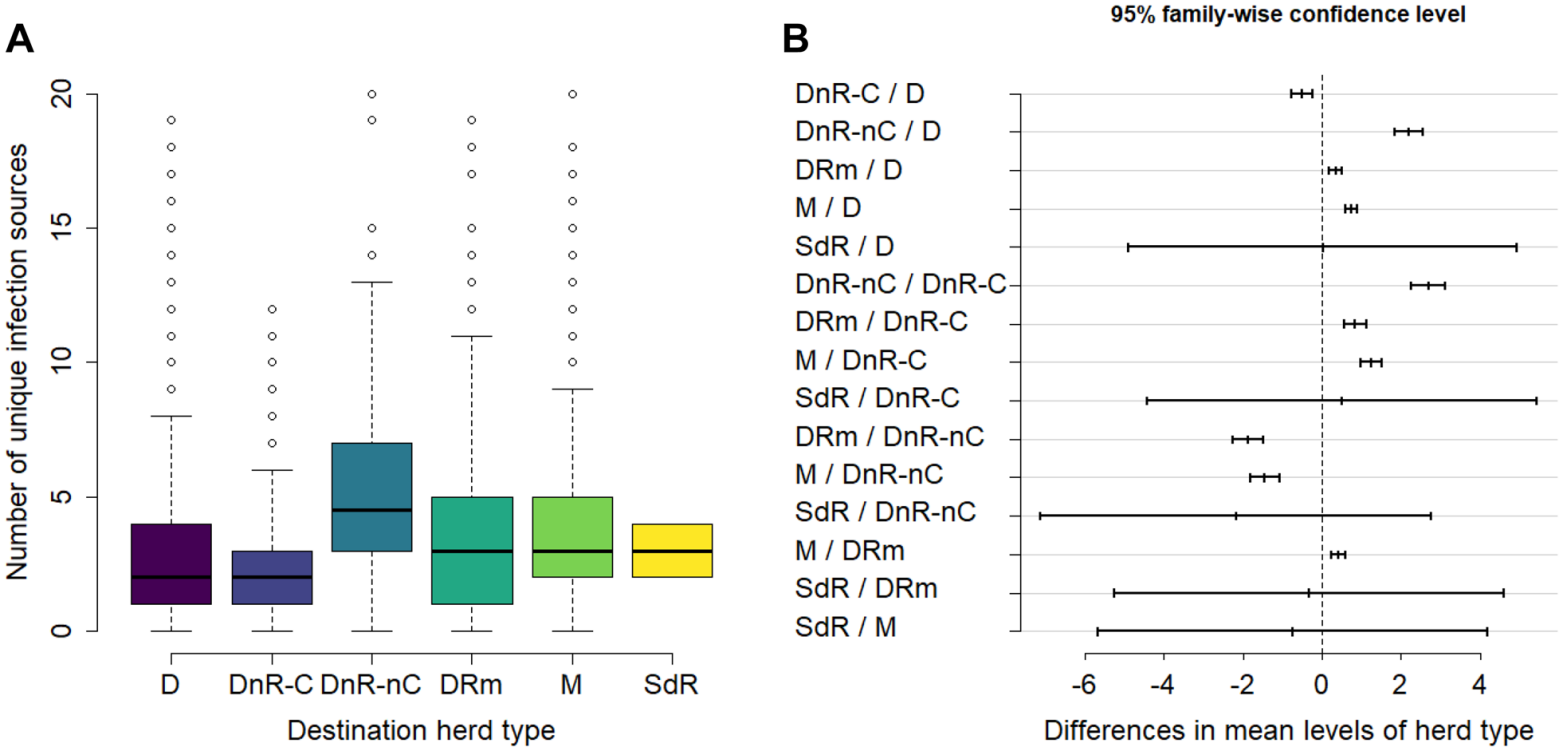
**The distribution of the number of unique infection sources per destination herd type.** A herd is counted as an infection source if it introduced an infected animal to the destination herd in at least one replicate. Herd types are described in Table 1. **A** Number of unique infection sources per herd summarized by herd type of destination herds. Each box contains values between the first and the third quartiles, the horizontal line corresponding to the median. Vertical lines outside the boxes extend to 10^th^ and 90^th^ percentiles. **B** Tukey test-95% family-wise confidence level for each combination of herd types. The figure presents the value of the difference between the means and their respective 95% CI. The vertical dashed line indicates the point where the difference in means is zero. For the definition of unique infection source see text.

Figure 8 presents the distribution of the number of unique infection sources per herd type. DnR-nC herds had significantly more unique infection sources compared to the other herd types. M herds had significantly more unique infection sources than D, DRm, and DnR-C. DRm herds had significantly more unique infection sources than D and DnR-C, and D herds had significantly more infection sources than DnR-C. So, for the mean number of unique infection sources: DnR-nC > M > DRm > D > DnR-C. Herds had on average 3.3 unique sources of infection, with a maximum of 20. Again the mean of herds classified as SdR was not significantly different from other herd types.

## Discussion

We used a stochastic individual-based transmission model to simulate *Map* spread between dairy cattle herds in Ireland, with the aim of investigating the effect of herd characteristics on the risk of becoming infected and spreading infection on a national scale. We showed that the probability of an infected animal being introduced into the herd increases both with increasing in-strength (the number of animals that enter a herd via trade) and in-degree (the number of herds from which animals are sourced). Furthermore, herds that both buy and sell a lot of animals pose the highest infection risk. Non-rearing dairy herds trade more animals and with more dairy herds than other herd types and could therefore play an important role in *Map* spread between dairy cattle herds.

We found that herds classified as non-rearing dairy herds infected on average more herds compared to other herd types. These herds (DnR-C and DnR-nC) do not rear their own female dairy calves. Calves are moved to contract rearing herds and pregnant heifers are bought back from the same contract rearing herd (DnR-C). Or cows are bred to beef bulls and the calves are sold, replacement heifers are bought from other herds (DnR-nC). The in-strength and out-strength (and to a lesser extent in-degree and out-degree) of these herd types was high compared to other herd types. DnR-C and DnR-nC herds also infected on average more herds compared to other herd types, and DnR-nC herds had the highest number of unique infection sources. Over 84% of the DnR-C and over 52% of the DnR-nC herds infected at least one other herd after they became infected themselves. Furthermore, 89% of the DnR-C herds and 40% of the DnR-nC herds had a probability greater than 97.5% of becoming infected during the 10-year simulation period. Because of these herd characteristics, it could be that these herd types play an important role in disease spread between herds. Although the trading structures are very different, pig herds in the pork supply chain with a high out-degree or long outgoing infection chain were of particular importance during an epidemic with regard to disease spread [45]. The in-going and out-going infection chains, while being complex to calculate for a national trade network of the size of the one considered in our study, could provide interesting insight in how much every herd type actually contributes to *Map* spread between dairy cattle herds.

We observed a strong positive correlation between in-degree and in-strength (0.80), meaning that herds that bought from a small number of other herds were also likely to buy a small total number of animals and vice-versa. Furthermore, we found a high correlation between in-strength and out-strength (0.72), meaning that herds that bought a lot of animals were likely to also sell a lot of animals, and vice-versa. Tratalos et al. [17], who analysed movements of all cattle in Ireland in 2016 including movements of males and animals of beef breed, also observed a strong correlation between in-degree and in-strength (0.88). However, there was little correlation between in- and out-strength (0.057). This could be because the trading pattern of female animals for dairy herds is different from the trading pattern of all animals (including males and animals of beef breed) for all cattle herds in Ireland which involves many different herd types. For most dairy herds trade is from herd to herd. These movements are all considered when calculating the in- and out-strength. In contrast, many beef herds sell directly to abattoirs, these movements were not considered for the calculations [17].

We found that the probability of a herd becoming infected during the 10-year simulation period increased rapidly with increasing number of animals purchased per year. Herds that bought more than 8 animals per year had, on average, a probability of over 95% of becoming infected. Also for France the probability of becoming infected increased with the number of animals purchased; French farms that bought three or more animals per year had a probability of over 50% of becoming infected within the 9-year simulation period [19]. However, buying an infected animal does not always imply disease persistence in the herd. For France, the probability of persistence decreases when the number of animals traded increases, i.e., a high turnover rate increases the probability of removing infected animals [19]. Further work could focus on investigating whether this phenomenon is also observed in Ireland.

We used polarity as a measure to investigate the probability of a herd becoming infected and thereafter being a risk of spreading infection to other herds. Results are presented separately for herds with an average in-strength of less than 4 and herds with an average in-strength of 4 or more. Herds with an average in-strength of less than 4 often escape infection and herds with an average in-strength of 4 or more have a high probability to become infected (Figures 4C and 5C). There is also a difference in mean polarity for herds with an in-strength of less than 4 and herds with an in-strength of 4 or more. Herds with an in-strength of less than 4 are generally sellers, while herds with an in-strength of 4 or more are generally wholesalers (herds that both buy and sell animals). For France, it was shown that wholesalers are more likely to spread infection compared to sellers or buyers [19]. In Ireland this is especially true for wholesalers with a high in-strength; because of the large number of animals going in and out of these herds, they not only have a high probability of becoming infected, but also have a high probability of spreading infection to other herds (Figure 7).

*Map* infection was very persistent, as shown by herd prevalence increasing over time for all replicates. Model predictions in dairy herds in France showed that, even with low herd prevalence and within-herd prevalence, extinction was impossible without intervention strategies [19]. However, herd prevalence could be lowered using combinations of control measures, for example by combining intervention strategies with risk based trading based on herd status to prevent *Map* introductions in free herds [20, 25]. In Ireland, herd prevalence was estimated at 28% in 2013–2014 [16]. In the French modelling study looking at risk-based trading [25], a much higher initial herd prevalence was assumed, but considering very low within-herd prevalence, most infected herds had a within-herd prevalence below the detection threshold. Another major difference between the two modelled regions lies in the number of trade movements, with many more trade movements in Ireland (2 304 149 for 13 353 herds) than in France (919 304 for 12 857 herds). It would, therefore, be interesting to investigate whether combining risk-based trading with intervention strategies would lead to similar results to those for France.

We included breeding bulls in the dataset because we wanted to account for the associated risk of *Map* introduction. After their introduction in the herd, bulls were assumed to behave like, and reside in the same location as, female animals of their age group. Transmission via semen [46–48], whether through natural service or artificial insemination, was not modelled as such. Beef bulls that were introduced came from herds outside of the metapopulation. The probability of a herd becoming infected increased with an increasing average number of bulls purchased per year. However, the average number of bulls purchased per year was not the best predictor for the probability of a herd to become infected, average in-strength being better. The number of bulls purchased per year was low (mean = 0.56) compared to the in-strength (mean = 6.37). Therefore, to reduce the risk of becoming infected, it is probably more effective to lower the number of female animals introduced from other herds than to stop using beef bulls for breeding.

The model is data-driven, meaning that herd-specific parameters such as initial herd size, exit rate, calves born over time and animal movements between herds were based on real trade data. An advantage of using real data is that there is a close resemblance to reality; actual herd demographics resemble simulated herd demographics, and herds interacting in reality are also interacting in the model. It was, therefore, possible to identify which herds with associated herd characteristics were most exposed to infection, had a higher chance of escaping it, or contributed most to *Map* spread. A disadvantage of using real data is that the simulated period and the number of herds modelled are constrained by the data, and simulations are based on past events. Farm numbers declined over the studied period [49, 50], and only herds with data over the whole period 2009–2018 were included in the model. Predicting animal movements based on trade patterns between herds would make it possible to simulate future dynamics as well. However, no method is available so far to predict animal movements at such a large scale (several thousand herds). In addition, obtaining accurate predictions might be difficult for some herds that have highly variable trade characteristics between years.

In the model, several assumptions are made. Validating the realism of these assumptions is not always easy because detailed data is not available. However, model components and the parameters used in the model are based on research, and updated when new information becomes available. For the within-herd part of the model, the transmission rates are the most uncertain. Two previous studies using a similar model, performed a sensitivity analysis on these parameters. For transmission within Irish dairy herds, only variation in the transmission rate parameter for the general indoor environment had an effect on prevalence over time, but the conclusion that the general indoor environment was the most important transmission route remained unchanged [10]. Similar results were found for transmission within French dairy herds, when transmission rate parameters were varied with 50% compared to the reference scenario [9]. For the between-herd part of the model, the only assumption that was made is that the herd and within-herd prevalence of herds outside of the metapopulation is the same as the herd and within-herd prevalence of herds within the metapopulation. Data to validate this assumption are lacking, and therefore it was simplest to assume that the average risk of introducing an infected animal from within and from outside of the metapopulation was the same.

Brock et al. [28] identified 17 different herd types in Ireland based on nine variables. Our focus was on dairy herds, and therefore, only herds belonging to one of the following six dairy herd types were included in the model. In typical dairy herds (D), the most common dairy herd type, most female dairy calves are reared to become replacement heifers and most of their male calves are sold at an early age. Therefore, there are almost no males between the age of 1 and 2 years in these herds. Mixed herds (M), the second most common dairy herd type, are characterised by having both milk and beef production activities; on average, these herd have half pure-bred dairy females and half cross-bred dairy and beef animals. For these mixed herds, even though there are two production types on the same farm, we assumed that the milk and beef production activities were managed in separate locations and that contact between dairy and beef animals was negligible. Therefore, we also neglected in- and out-movements related to the beef production activity. The third most common herd type is dairy herds that also rear their male calves (DRm). Similar to typical dairy herds (D), female dairy calves are reared to become replacement heifers, however, in DRm herds the males are kept, resulting in a high proportion of male animals between the age of 1 and 2 years. In the model, only data on female animals was included (with the exception of some data on breeding bulls), therefore, data on male animals in DRm herds was not included. Since the males are sold as young stock before the age of 104 weeks for fattening [51], we assumed that the probability that they are shedding *Map* substantially in the presence of young dairy female calves is low, and therefore, their contribution to *Map* spread is minimal. Slightly less prevalent were the non-rearing dairy herds (DnR-C and DnR-nC). These herds sell most of their calves, with female dairy calves being moved to external contract rearing herds (e.g., herds that rear dairy females (SdR)). The difference between DnR-C herds and DnR-nC herds is that in DnR-C herds the female calves return to their birth herd as pregnant heifers (contract rearing), whereas in DnR-nC herds there is no contract rearing and replacement animals are bought from other herds with a surplus of cows or pregnant heifers. In SdR herds, female dairy calves are reared and inseminated before returning to their birth herd (the DnR-C herds). In these herds, animals are usually of young age. Since herds with less than five adults were too small to be taken into account by the model, only two SdR herds were included in the metapopulation.

Non-dairy herds were connected to the dairy herds as well. However, 63% of the movements were from a dairy herd in the metapopulation to a herd outside of the metapopulation. Only 20% of the movements were from a herd outside of the metapopulation to a herd in the metapopulation. Brock et al. [51] investigated transport flows per herd type for Ireland. The majority of the movements to dairy herds came from store herds. In our manuscript, only two store herds (SdR) were included, mainly because in store herds often only young animals are present (i.e., young female dairy calves are introduced, reared and inseminated, before being returned to their birth herd). Most store herds were excluded from the metapopulation based on the criteria that at least five adult animals needed to be present in the herd. However, movements from these herds into the metapopulation were still simulated. The risk of introducing an infected animal through such a movement was drawn from a distribution that corresponded to the proportion of animals in each health state in the same age group within the entire metapopulation. So the risk of transmission was accounted for. Unfortunately, it was not possible to include non-dairy herd types because the model was specifically designed to represent dairy herd management.

In the model, we assumed that herd management was similar for all farms, even though the characteristics of the herd types, especially with regard to which age groups are present, are different. In the model, animals were divided into age classes, each with their own properties, and therefore all herds could be modelled in the same way. For example, for all herds we assumed that cows would go to pasture in the beginning of March, whereas calves stay indoors to the end of April. Thus, properties between age classes were different but for a given age class properties were the same across farm types.

We have shown that herds that buy many animals from a lot of different source herds have the highest probability of becoming infected, whereas herds that buy only a few animals from a few sources have the highest probability of escaping infection. Furthermore, on average, herds classified as non-rearing dairy herds are at greatest risk of infecting other herds, compared to other herd types. These findings could be used to identify herds on which the control programme could particularly focus, including those at highest risk of being infected and those at highest risk of spreading infection to other herds. For example, by combining intervention strategies with risk based trading, there is the possibility to prevent *Map* introduction into herds that are currently free of infection.

## Supplementary Information

**Additional file 1. List of dairy breeds and table containing model parameters.**

**Additional file 2. Schematic representation of the within-herd *Map* transmission model.**

**Additional file 3. Simulated versus observed herd sizes, when simulations are based on seasonal exit rates.**

**Additional file 4. Visualisation of the range of the in-degree, out-degree, in-strength, and out-strength span per herd.**

**Additional file 5. Explanation of the importance of choosing the right herds to seed with infection at the start of the simulations.** Showing that the results obtained are affected by which herds are selected to be initially infected.

**Additional file 6. Probability distribution from which the initial within-herd prevalence was sampled.**

## Data Availability

The datasets used and/or analysed during the current study are available from the corresponding author on reasonable request.

## References

[1] Garcia AB, Shalloo L (2015). Invited review: The economic impact and control of paratuberculosis in cattle. J Dairy Sci.

[2] Windsor PA, Whittington RJ (2010). Evidence for age susceptibility of cattle to Johne’s disease. Vet J.

[3] Whitlock RH, Buergelt C (1996). Preclinical and clinical manifestations of paratuberculosis (including pathology). Vet Clin N Am Food Anim Pract.

[4] Mitchell RM, Schukken Y, Koets A, Weber M, Bakker D, Stabel J, Whitlock RH, Louzoun Y (2015). Differences in intermittent and continuous fecal shedding patterns between natural and experimental *Mycobacterium avium* subspecies *paratuberculosis* infections in cattle. Vet Res.

[5] Barkema HW, Orsel K, Nielsen SS, Koets AP, Rutten VPMG, Bannantine JP, Keefe GP, Kelton DF, Wells SJ, Whittington RJ, Mackintosh CG, Manning EJ, Weber MF, Heuer C, Forde TL, Ritter C, Roche S, Corbett CS, Wolf R, Griebel PJ, Kastelic JP, De Buck J (2018). Knowledge gaps that hamper prevention and control of *Mycobacterium avium* subspecies *paratuberculosis* infection. Transbound Emerg Dis.

[6] More SJ, Cameron AR, Strain S, Cashman W, Ezanno P, Kenny K, Fourichon C, Graham D (2015). Evaluation of testing strategies to identify infected animals at a single round of testing within dairy herds known to be infected with *Mycobacterium avium* ssp. *paratuberculosis*. J Dairy Sci.

[7] Marquetoux N, Heuer C, Wilson P, Ridler A, Stevenson M (2016). Merging DNA typing and network analysis to assess the transmission of paratuberculosis between farms. Prev Vet Med.

[8] Sweeney RW, Collins MT, Koets AP, Mcguirk SM, Roussel AJ (2012). Paratuberculosis (Johne’s disease) in cattle and other susceptible species. J Vet Intern Med.

[9] Marcé C, Ezanno P, Seegers H, Pfeiffer DU, Fourichon C (2011). Predicting fadeout versus persistence of paratuberculosis in a dairy cattle herd for management and control purposes: a modelling study. Vet Res.

[10] Biemans F, Ben Romdhane R, Gontier P, Fourichon C, Ramsbottom G, More SJ, Ezanno P (2021). Modelling transmission and control of *Mycobacterium avium* subspecies *paratuberculosis* within Irish dairy herds with compact spring calving. Prev Vet Med.

[11] Ramsbottom G, Horan B, Berry DP, Roche JR (2015). Factors associated with the financial performance of spring-calving, pasture-based dairy farms. J Dairy Sci.

[12] Butler ST, Shalloo L, Murphy JJ (2010). Extended lactations in a seasonal-calving pastoral system of production to modulate the effects of reproductive failure. J Dairy Sci.

[13] Gavey L, Citer L, More SJ, Graham D (2021). The Irish Johne’s Control Programme. Front Vet Sci.

[14] Jordan AG, Citer LR, McAloon CG, Graham DA, Sergeant ESG, More SJ (2020). Johne’s disease in Irish dairy herds: considerations for an effective national control programme. Ir Vet J.

[15] Good M, Clegg T, Sheridan H, Yearsely D, O’Brien T, Egan J, Mullowney P (2009). Prevalence and distribution of paratuberculosis (Johne’s disease) in cattle herds in Ireland. Ir Vet J.

[16] McAloon CG, Doherty ML, Whyte P, O’Grady L, More SJ, Locksley L, McV M, Good M, Mullowney P, Strain S, Green MJ (2016). Bayesian estimation of prevalence of paratuberculosis in dairy herds enrolled in a voluntary Johne’s Disease Control Programme in Ireland. Prev Vet Med.

[17] Tratalos JA, Madden JM, McGrath G, Graham DA, Collins ÁB, More SJ (2020). Spatial and network characteristics of Irish cattle movements. Prev Vet Med.

[18] Ezanno P, Andraud M, Beaunée G, Hoch T, Krebs S, Rault A, Touzeau S, Vergu E, Widgren S (2020). How mechanistic modelling supports decision making for the control of enzootic infectious diseases. Epidemics.

[19] Beaunée G, Vergu E, Ezanno P (2015). Modelling of paratuberculosis spread between dairy cattle farms at a regional scale. Vet Res.

[20] Beaunée G, Vergu E, Joly A, Ezanno P (2017). Controlling bovine paratuberculosis at a regional scale: towards a decision modelling tool. J Theor Biol.

[21] Rossi G, De Leo GA, Pongolini S, Natalini S, Zarenghi L, Ricchi M, Bolzoni L (2017). The potential role of direct and indirect contacts on infection spread in dairy farm networks. PLoS Comput Biol.

[22] Knific T, Ocepek M, Kirbiš A, Lentz HHK (2020). Implications of cattle trade for the spread and control of infectious diseases in Slovenia. Front Vet Sci.

[23] Knific T, Kirbiš A, Gethmann JM, Prezelj J, Krt B, Ocepek M (2022). Modeling paratuberculosis transmission in a small dairy herd typical of Slovenia suggests that different models should be used to study disease spread in herds of different sizes. Animals.

[24] Camanes G, Joly A, Fourichon C, Ben Romdhane R, Ezanno P (2018). Control measures to prevent the increase of paratuberculosis prevalence in dairy cattle herds: an individual-based modelling approach. Vet Res.

[25] Ezanno P, Arnoux S, Joly A, Vermesse R (2022). Rewiring cattle trade movements helps to control bovine paratuberculosis at a regional scale. Prev Vet Med.

[26] Tratalos JA, Graham DA, More SJ (2017). Patterns of calving and young stock movement in Ireland and their implications for BVD serosurveillance. Prev Vet Med.

[27] DAFM (2019) AIM bovine statistics report 2019. https://assets.gov.ie/101723/4be97151-4365-415d-a0e2-87c033ae279e.pdf. Accessed 02 June 2022.

[28] Brock J, Lange M, Tratalos JA, More SJ, Graham DA, Guelbenzu-Gonzalo M, Thulke H-H (2021). Combining expert knowledge and machine-learning to classify herd types in livestock systems. Sci Rep.

[29] Marcé C, Guatteo R, Bareille N, Fourichon C (2010). Dairy calf housing systems across Europe and risk for calf infectious diseases. Animal.

[30] Marcé C, Ezanno P, Seegers H, Pfeiffer DU, Fourichon C (2011). Within-herd contact structure and transmission of *Mycobacterium avium* subspecies *paratuberculosis* in a persistently infected dairy cattle herd. Prev Vet Med.

[31] Berry D, Buckley F, Butler S, Kennedy E, Ramsbottom G, Donworth J (2016) Section 7-Dairy breeding. In: Teagasc dairy manual. https://www.teagasc.ie/media/website/publications/2016/Dairy-Manual-Section7.pdf. Accessed 02 June 2022

[32] Giese SB, Ahrens P (2000). Detection of *Mycobacterium avium* subsp. *paratuberculosis* in milk from clinically affected cows by PCR and culture. Vet Microbiol.

[33] Whittington RJ, Marshall DJ, Nicholls PJ, Marsh IB, Reddacliff LA (2004). Survival and dormancy of *Mycobacterium avium* subsp. *paratuberculosis* in the environment. Appl Environ Microbiol.

[34] Moslonka-Lefebvre M, Gilligan CA, Monod H, Belloc C, Ezanno P, Filipe JAN, Vergu E (2016). Market analyses of livestock trade networks to inform the prevention of joint economic and epidemiological risks. J R Soc Interface.

[35] McAloon CG, Doherty ML, Whyte P, O’Grady L, More SJ, Messam LLM, Good M, Mullowney P, Strain S, Green MJ (2016). Bayesian estimation of prevalence of paratuberculosis in dairy herds enrolled in a voluntary Johne’s Disease Control Programme in Ireland. Prev Vet Med.

[36] R Core Team (2021) R: a language and environment for statistical computing. https://www.r-project.org/. Accessed 02 June 2022

[37] Wickham H (2016). ggplot2: Elegant Graphics for data analysis.

[38] Wilke CO (2020) ggridges: ridgeline plots in “ggplot2.”. https://cran.r-project.org/package=ggridges. Accessed 02 June 2022

[39] Garnier S (2018) Viridis: default color maps from “matplotlib.”. https://cran.r-project.org/package=viridis. Accessed 02 June 2022

[40] Peterson BG, Carl P (2020) PerformanceAnalytics: econometric tools for performance and risk analysis. https://cran.r-project.org/package=PerformanceAnalytics. Accessed 02 June 2022

[41] Hothorn T, Bretz F, Westfall P (2008). Simultaneous inference in general parametric models. Biometr J.

[42] Wickham H (2007). Reshaping data with the reshape package. J Stat Softw.

[43] Wickham H, François R, Henry L, Müller K (2021) dplyr: a grammar of data manipulation. https://cran.r-project.org/package=dplyr. Accessed 02 June 2022

[44] Wickham H (2021) tidyr: Tidy Messy Data. https://cran.r-project.org/package=tidyr. Accessed 02 June 2022

[45] Büttner K, Krieter J, Traulsen A, Traulsen I (2013). Efficient interruption of infection chains by targeted removal of central holdings in an animal trade network. PLoS ONE.

[46] Larsen AB, Kopecky KE (1970). Mycobacterium paratuberculosis in reproductive organs and semen of bulls. Am J Vet Res.

[47] Ayele W, Machackova M, Pavlik I (2001). The transmission and impact of paratuberculosis infection in domestic and wild ruminants. Vet Med.

[48] Khol JL, Kralik P, Slana I, Beran V, Aurich C, Baumgartner W, Pavlik I (2010). Consecutive excretion of *Mycobacterium avium* subspecies *paratuberculosis* in semen of a breeding bull compared to the distribution in feces, tissue and blood by IS900 and F57 quantitative real-time PCR and culture examinations. J Vet Med Sci.

[49] CSO (2012) Census of Agriculture 2010—final results. https://www.cso.ie/en/media/csoie/releasespublications/documents/agriculture/2010/full2010.pdf. Accessed 02 June 2022

[50] Shalloo L, Connor DO, Cele L, Thorne F, Egan M (2020) An analysis of the Irish dairy sector post quota. https://www.teagasc.ie/publications/2020/an-analysis-of-the-irish-dairy-sector-post-quota-.php. Accessed 02 June 2022

[51] Brock J, Lange M, Tratalos JA, More SJ, Guelbenzu-Gonzalo M, Graham DA, Thulke H-H (2021). A large-scale epidemiological model of BoHV-1 spread in the Irish cattle population to support decision-making in conformity with the European Animal Health Law. Prev Vet Med.

