# Supplementary material for: Modelling transmission of Mycobacterium avium subspecies paratuberculosis between Irish dairy cattle herds
Source: Vet Res. 2022 Jun 22;53:45. doi: 10.1186/s13567-022-01066-5 (PMC9215035; doi:10.1186/s13567-022-01066-5)
Supplement: Supplementary file 1 — Additional file 1. List of dairy breeds and table containing model parameters. [file 13567_2022_1066_MOESM1_ESM.docx]

**Additional file 1**

*Data selection*

An animal was classified as dairy when it was of one of the following breeds: Armoricaine, Angler, Ayrshire, Blue Albion, Bretonne Pie-noire, Brown Swiss, Frisona Espagnola, Flekvieh, Holstein/Friesian, Groninger Blaarkop, Guernsey, Jersey, Lithuanian Black and White, Malkekorthorn, Blended Milking Shorthorn, Normande, Norwegian Red, Danish Red, Reggiana, Swedish red, or Valdostana Nera.

*Model parameters*

Table S1.1 presents the details of all model parameters.

**Table S1.1. Model parameters.**

| **Value** | | **Parameter definition** | **Source** |
| --- | --- | --- | --- |
| 14 | Weaning age, when an unweaned calf becomes a weaned calf (weeks) | | ^a^ |
| 104 | Age at first calving, when a bred heifer becomes a cow (weeks) | | [20] |
| 5 | Maximum number of parities | | ^b^ |
| 16 | Start grazing calves (week number) | | [46] |
| 11 | Start grazing young heifers (week number) | | [46] |
| 9 | Start grazing cows (week number) | | [46, 47] |
| 45 | End grazing all animals (week number) | | [46, 47] |
| 5.0 | Colostrum consumed by newborn calves for 5.5 days (L/calf/day) | | _­_[20] |
| 7.0 | Milk consumed by unweaned calves after first 5.5 days (L/calf/day) | | [20] |
| 0.4 | Faeces produced by newborn and unweaned calves (kg/day) | | ^a^ |
| 4.1 | Faeces produced by a weaned calf (kg/day) | | ^a^ |
| 7.5 | Faeces produced by a young heifer (kg/day) | | ^a^ |
| 22.5 | Faeces produced by bred heifers and cows (kg/day) | | ^a^ |
| 0.92 | | Reduction factor for milk production, I_L_^c^ cows | [48, 49] |
| 0.89 | | Reduction factor for milk production, I_M_^c^ cows | [48, 49] |
| 0.75 | | Reduction factor for milk production, I_H_^c^ cows | [48, 49] |
| 0.400 | | Weekly mortality rate of *Map* in an indoor environment | [23] |
| 0.071 | | Weekly mortality rate of *Map* on pasture | [50] |
| 0.83 | | Reduction factor quantity *Map* after additional cleaning pens once | ^a^ |
| $1\times{10}^{6}$ | | Infectious dose of *Map* | [51] |
| $9.5\times{10}^{-7}\times7$ | | Transmission rate parameter for general indoor environment (week^-1^) | [52] |
| $5.0\times{10}^{-5}\times7$ | | Transmission rate parameter for local indoor environment (week^-1^) | [52] |
| $5.0\times{10}^{-6}\times7$ | | Transmission rate parameter for local pasture environment (week^-1^) | ^b^ |
| $5.0\times{10}^{-4}\times7$ | | Transmission rate parameter for milk/colostrum (week^-1^) | ^b^ |
| 0.149 | | Probability of in utero transmission for I_T_^c^, I_L_, and I_M_ cows | [53, 54] |
| 0.650 | | Probability of in utero transmission for I_H_ cows | [53, 54] |

^a^ Expert opinion

^b^ Model assumption

^c^ Transiently infectious (I_T_), latently infected (I_L_), moderately infectious (I_M_), highly infectious (I_H_)
