# Supplementary material for: Modelling transmission of Mycobacterium avium subspecies paratuberculosis between Irish dairy cattle herds
Source: Vet Res. 2022 Jun 22;53:45. doi: 10.1186/s13567-022-01066-5 (PMC9215035; doi:10.1186/s13567-022-01066-5)
Supplement: Supplementary file 2 — Additional file 2. Schematic representation of the within-herd Map transmission model. [file 13567_2022_1066_MOESM2_ESM.docx]

**Additional file 2**

**
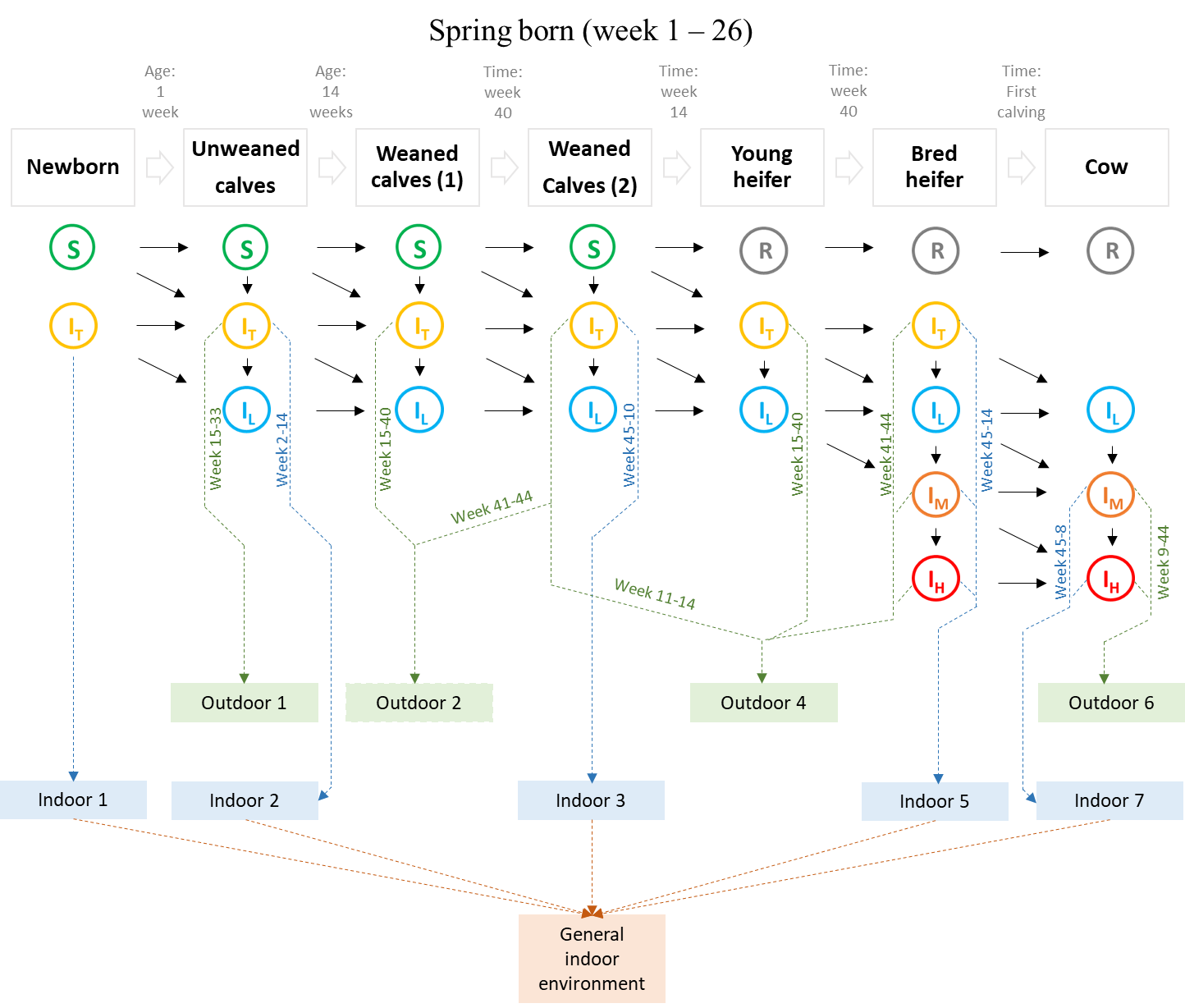
**

**
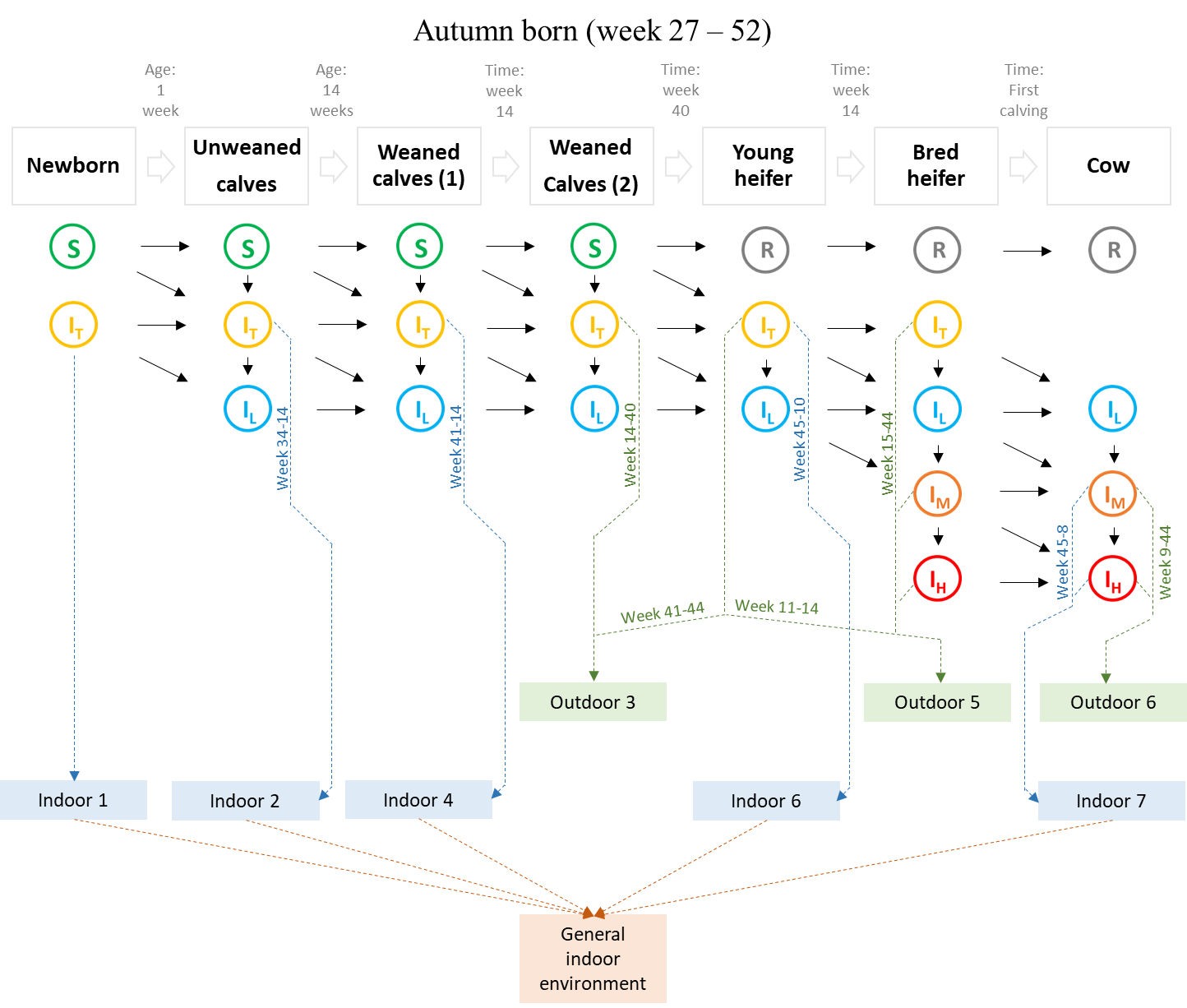
**

**Figure S2.1. Schematic representation of the within-herd paratuberculosis transmission model.** A distinction was made between spring born (top) and autumn born (bottom) calves. Health states include susceptible (S), resistant (R), transiently infectious (I_T_), latently infected (I_L_), moderately infectious (I_M_), and highly infectious (I_H_). Transitions between health states are represented by solid lines. Infectious animals shed *Mycobacterium avium* subspecies *paratuberculosis* (*Map*) in their respective local indoor/outdoor environments (dashed lines). The summed quantity of *Map* in the local indoor environments makes up the amount present in the general indoor environment.
