# Supplementary material for: Modelling transmission of Mycobacterium avium subspecies paratuberculosis between Irish dairy cattle herds
Source: Vet Res. 2022 Jun 22;53:45. doi: 10.1186/s13567-022-01066-5 (PMC9215035; doi:10.1186/s13567-022-01066-5)
Supplement: Supplementary file 3 — Additional file 3. Simulated versus observed herd sizes, when simulations are based on seasonal exit rates. [file 13567_2022_1066_MOESM3_ESM.docx]

**Additional file 3**

*Observed versus modelled herd size*

Observed and modelled herd size were compared 20 times over the ten-year simulated period, at the 1^st^ of January and the 1^st^ of July of every year. We used this comparison to investigate whether the use of yearly, seasonal, or monthly exit rates resulted in a better agreement between actual and simulated herd size. For every herd, agreement between observed and modelled herd size was assessed by calculating the number of times the modelled herd size did not fall in the <0.75 * Herd size data – 1.25 * Herd size data> range. With seasonal exit rates (i.e., exit rates over a three-month period; January – March, April – June, July – September, October – December), for 9.9% of the herds the modelled herd size was outside of this range more than four times (out of twenty). With yearly exit rates, for 14.3% of the herds the modelled herd size was outside of this range more than four times. And with monthly exit rates, for 16.9% of the herds the modelled herd size was outside of this range more than four times. Thus, the use of seasonal exit rates resulted in the best agreement between observed and modelled herd size compared to the use of yearly or monthly exit rates. Figure A3.1 shows the modelled herd size (herd size in simulations) versus herd size of data when seasonal exit rates are used.


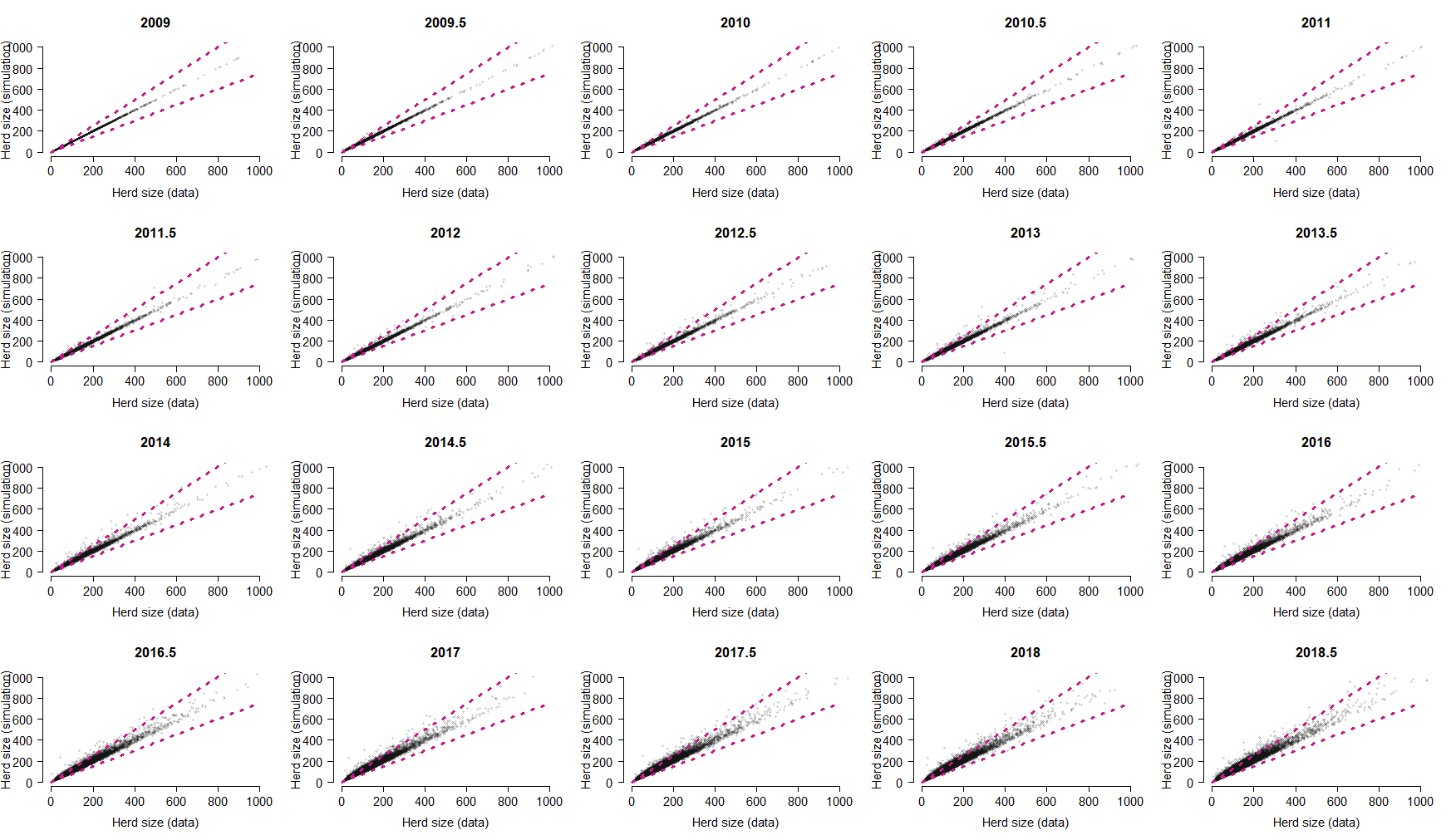


**Figure S3.1. Simulated versus observed herd sizes, when simulations are based on seasonal culling rates.** Herd size was assessed on the 1^st^ of January (year) and the 1^st^ of July (year.5) of every year. The pink dotted lines show: “Herd size simulation = Herd size data * 0.75” and “Herd size simulation = Herd size data * 1.25”.
