# Supplementary material for: Modelling transmission of Mycobacterium avium subspecies paratuberculosis between Irish dairy cattle herds
Source: Vet Res. 2022 Jun 22;53:45. doi: 10.1186/s13567-022-01066-5 (PMC9215035; doi:10.1186/s13567-022-01066-5)
Supplement: Supplementary file 4 — Additional file 4. Visualisation of the range of the in-degree, out-degree, in-strength, and out-strength span per herd. [file 13567_2022_1066_MOESM4_ESM.docx]

**Additional file 4**


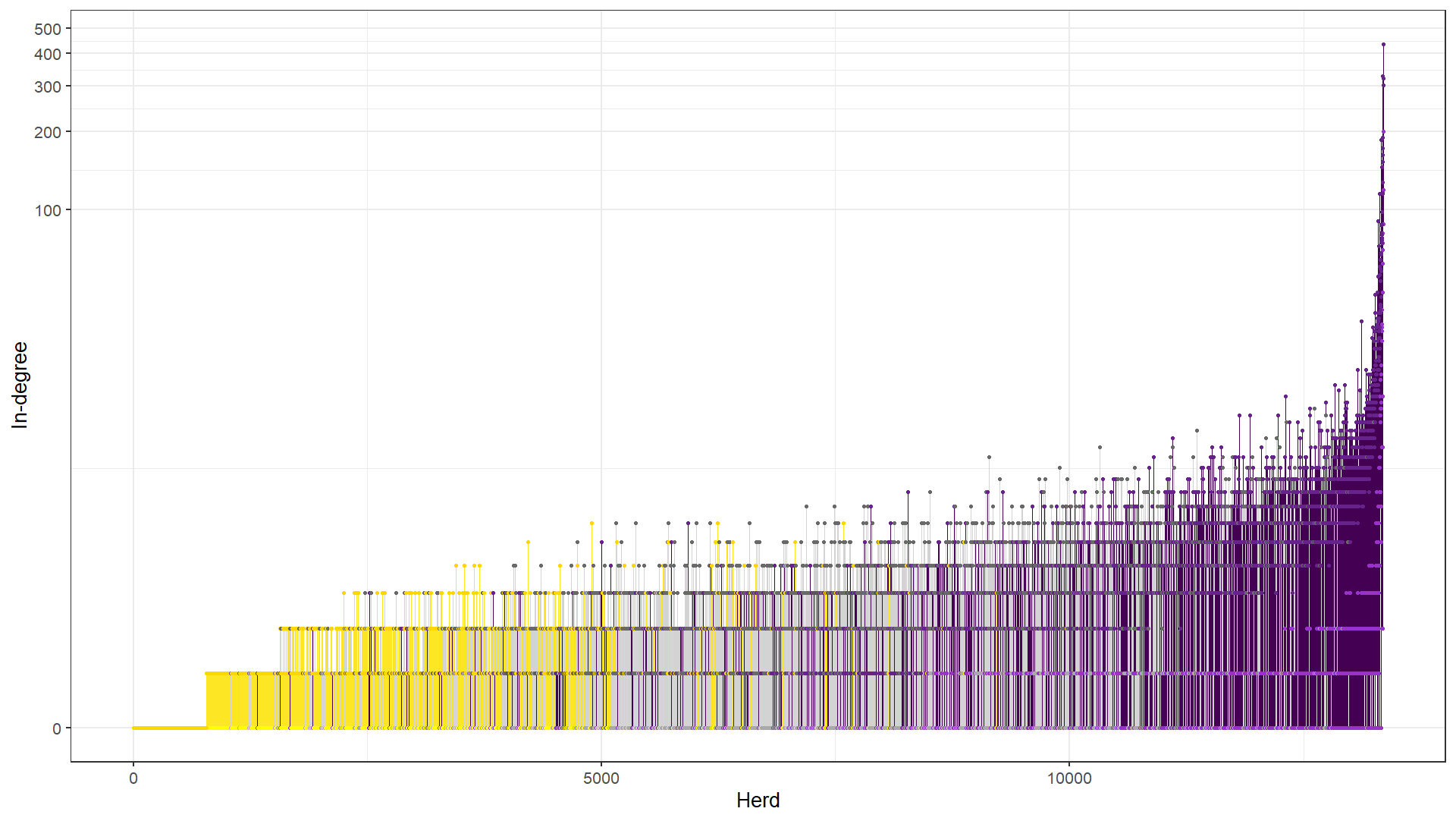

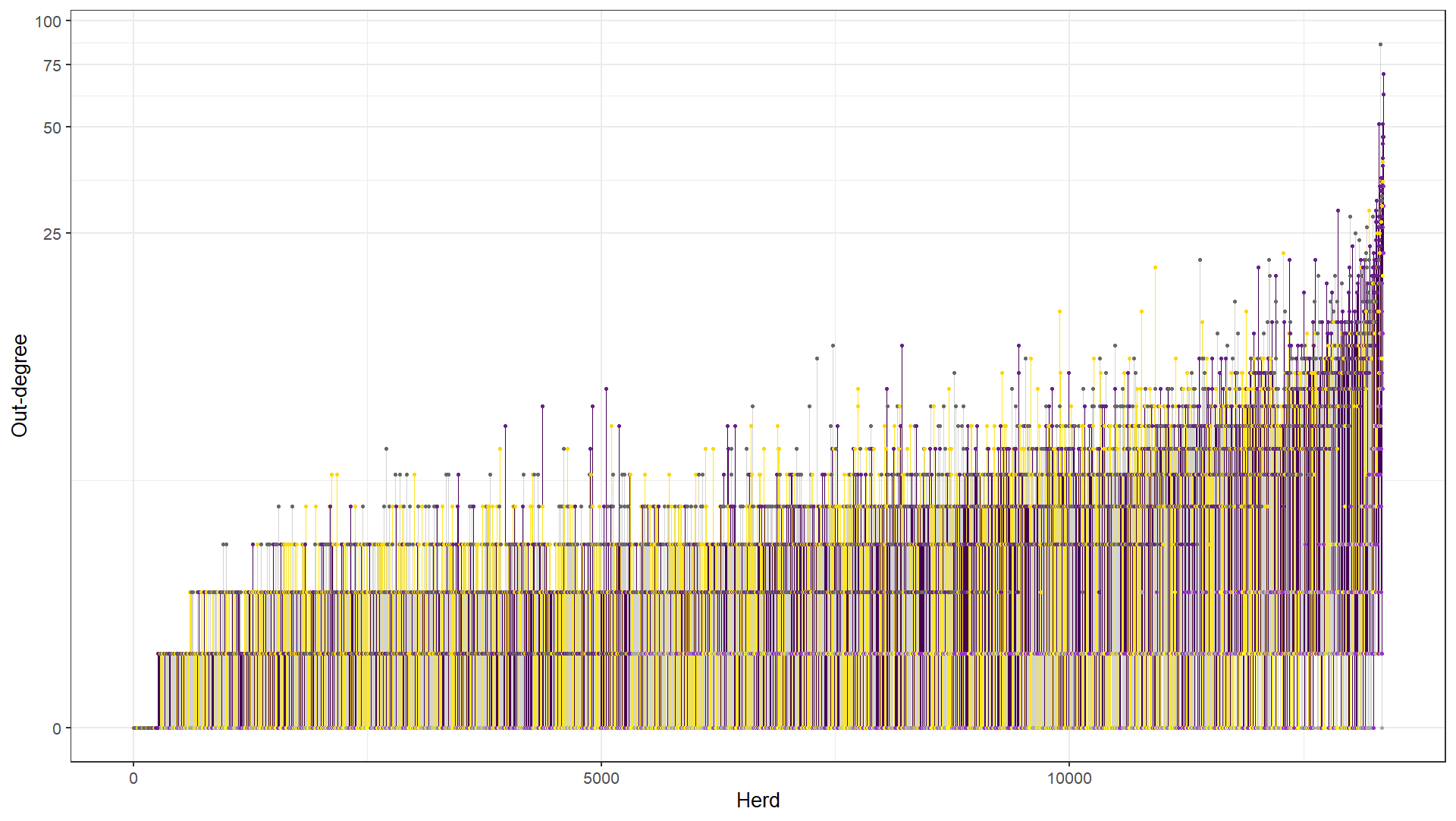


**Figure S4.1. Visualisation of the range of the in-degree (top) and out-degree (bottom) span per herd.** For every herd the lowest and highest yearly degree are represented by a dot and the lines connecting the dots represent the full range. Herds are ordered by their average degree, with the herd with the lowest average degree on the left and the herd with the highest degree on the right. The 30% of herds that had the lowest probability of being infected at the end of the ten-year simulated period given that they were not infected at the start when 25% of the herds were infected at random (see Additional file 5) are in yellow, the 30% of herds with the highest probability of being infected at the end of the ten-year simulated period given that they were not infected at the start are in purple, and herds not belonging to either of these categories are in grey.
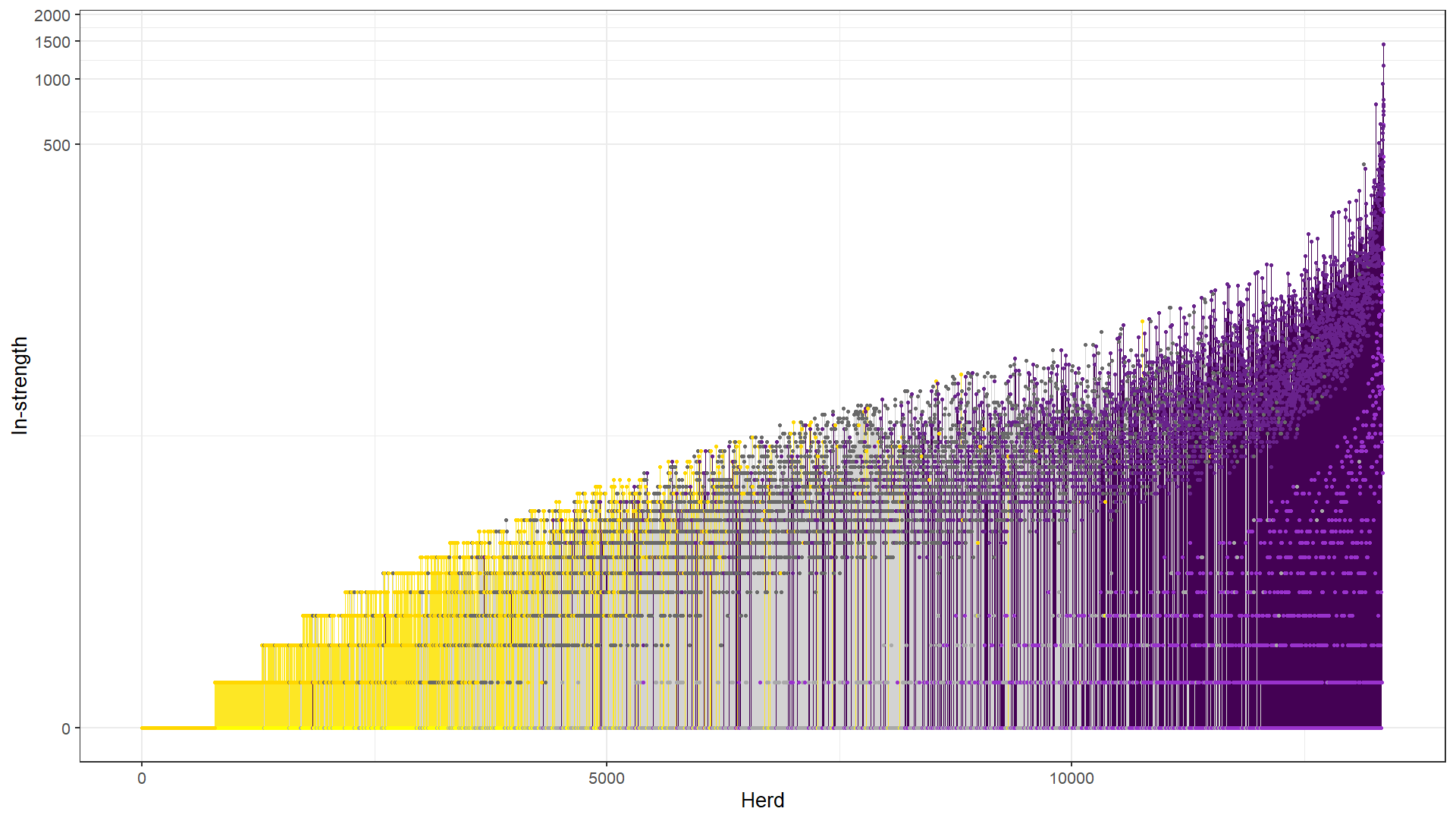

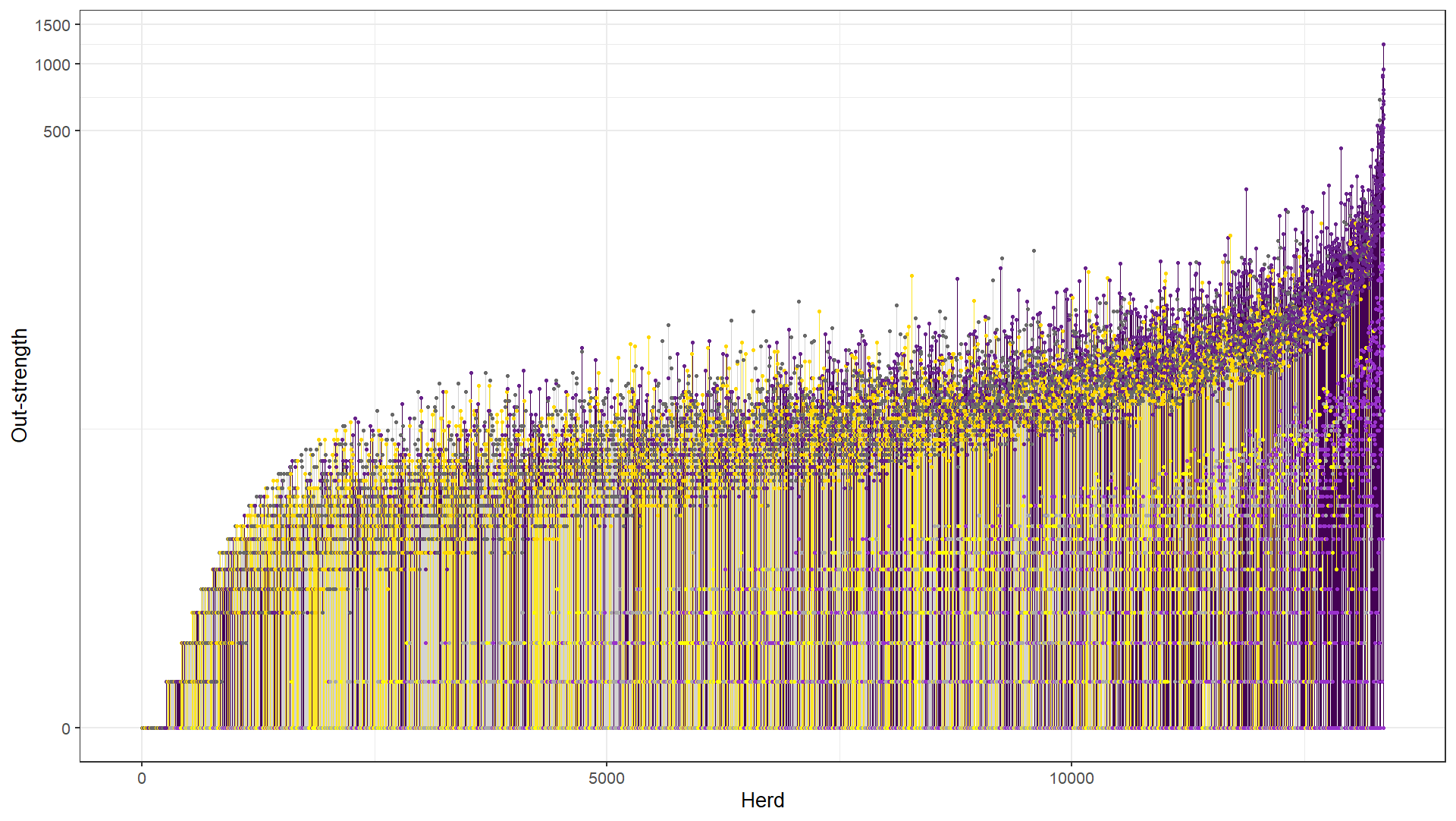


**Figure S4.2. Visualisation of the range in in-strength (top) and out-strength (bottom) per herd.** For every herd the lowest and highest yearly strength are represented by a dot and the lines connecting the dots represent the full range. Herds are ordered by their average strength, with the herd with the lowest average strength on the left and the herd with the highest strength on the right. The 30% of herds that had the lowest probability of being infected at the end of the ten-year simulated period given that they were not infected at the start when 25% of the herds were infected at random (see Additional file 5) are in yellow, the 30% of herds with the highest probability of being infected at the end of the ten-year simulated period given that they were not infected at the start are in purple, and herds not belonging to either of these categories are in grey.
