# Supplementary material for: Modelling transmission of Mycobacterium avium subspecies paratuberculosis between Irish dairy cattle herds
Source: Vet Res. 2022 Jun 22;53:45. doi: 10.1186/s13567-022-01066-5 (PMC9215035; doi:10.1186/s13567-022-01066-5)
Supplement: Supplementary file 5 — Additional file 5. Explanation of the importance of choosing the right herds to seed with infection at the start of the simulations. Showing that the results obtained are affected by which herds are selected to be initially infected. [file 13567_2022_1066_MOESM5_ESM.docx]

**Additional file 5 The importance of the starting conditions**

Which herds are chosen as the primary infected herds at the start has an effect on the results. To assess this effect, we compared two scenarios: 1) choose herds to infect at initialization from the 30% most likely to be infected (as presented in the main manuscript), and 2) choose herds to infect at initialization from the 30% least likely to be infected. To determine which herds belonged to these categories, we first assessed for each herd the probability of being infected after ten years given that the herd was not initially infected. For 1000 replicates, 25% of the herds were randomly chosen to be infected with a within-herd prevalence of 0–70% drawn from a Gaussian distribution N(-0.42,0.12); only values in the range from >0 to 0.7 were used (Additional file 6). After ten years of simulated *Map* transmission, the probability of being infected was calculated. From this the 30% of herds most likely to be infected and the 30% least likely to be infected were determined. Next, for both scenarios, about 3,338 herds (25% of the total number of herds) were chosen and initialized with a within-herd prevalence of 0-70%, again drawn from the aforementioned distribution.

Figure S5.1 presents the distribution of the probability of being infected after ten years of simulation given that a herd was not part of the 25% initially infected herds. The 30% of herds most likely to be infected had a >73.3% probability of being infected when herds were initialized at random. For 482 herds the probability of being infected was 100%. The 30% of herds least likely to be infected had a <22.4% probability of being infected. For 851 herds the probability of being infected was 0%.

Figure S5.2 presents the distribution of herds classified as most likely or least likely to be infected among herd types. The percentage classified as most likely to be infected was 24.9% for typical dairy herds, 88.7% for dairy no rearing – contract herds, 38.2% for dairy no rearing – no contract herds, 22.8% for dairy herds that are also rearing male calves, 30.0% for mixed herds, and 100% for herds that rear dairy females. The percentage classified as least likely to be infected was 36.3% for dairy herds, 0.9% for dairy no rearing – contract herds, 15.0% for dairy no rearing – no contract herds, 33.2% for dairy herds that are also rearing male calves, 24.8% for mixed herds, and no herds that rear dairy females.


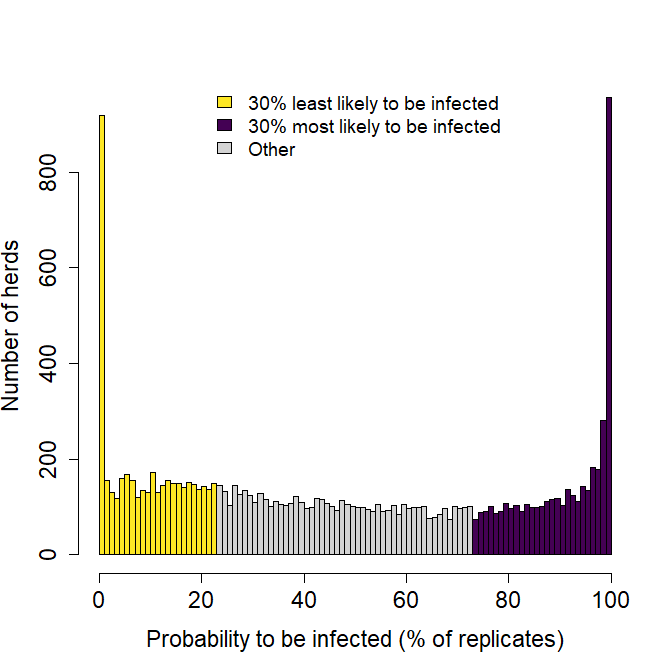


**Figure S5.1. Distribution of the probability of being infected.** Probability of being infected is defined as the probability that there are infected animals present in a herd at the end of the ten-year simulated period given that the herd was not part of the 25% of herds which were initially infected.


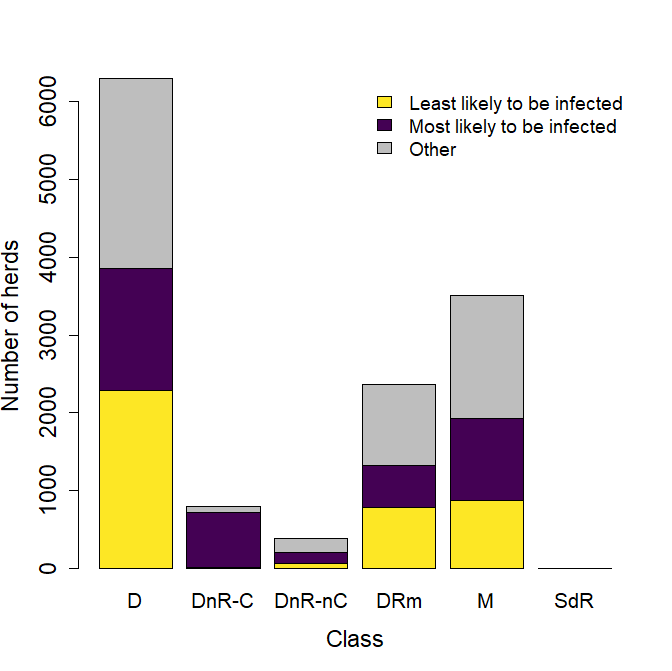


**Figure S5.2. Distribution of herds classified as most or least likely to be infected among herd types.** Herd types are typical dairy (D), dairy no rearing – contract (DnR-C), dairy no rearing – no contract (DnR-nC), dairy also rearing male calves (DRm), mixed (M), and herds that rear dairy females (SdR). Herds were classified as most likely to be infected when they belonged to the 30% of herds with the highest probability of being infected at the end of the ten-year simulated period given that they were not infected at the start (purple). Herds were classified as least likely to be infected when they belonged to the 30% of herds that had the lowest probability of being at the end of the ten-year simulated period given that they were not infected at the start (yellow). Herds not belonging to either of these categories are in grey.

Figure S.5.3 presents the herd prevalence over time for all replicates. In 1000 replicates a random 25% of the herds were seeded with infection, in 295 replicates part of the 30% most likely herds were seeded with infection, and in 100 replicates part of the 30% least likely herds were seeded with infection. Which herds are initially infected has an effect on the results; when a random sample of herds was chosen to be initially infected, herd prevalence after ten years of simulation was on average 58.7%. When a proportion of the most likely herds was initially infected, herd prevalence after ten years of simulation was on average 49.9%. However, when a proportion of the least likely herds was initially infected, herd prevalence after ten years was on average 68.0%.

Figure S5.4 presents the percentage of least (most) likely herds infected over time when the least (most) likely herds were initially infected, and the percentage of least (most) likely herds infected over time when the most (least) likely herds were initially infected. When the most likely herds were seeded with infection, 97.9% of the seeded herds were still infected after 10 years (Figure S5.4A purple), and only 9.1% of the least likely herds were infected (Figure S5.4B yellow). In contrast, when the least likely herds were initially infected, 86.3% of the seeded herds were still infected after 10 years (Figure S5.4A yellow), but also 89.2% of the most likely herds were infected (Figure S5.4B purple). This shows that when the most likely herds are seeded with infection, the majority of the least likely herds will not be infected, but when the least likely herds are seeded with infection, the majority of the most likely herds will still be infected. Therefore, seeding the least likely herds with infection might lead to results (e.g., herd prevalence) that are not likely to be observed in the field.


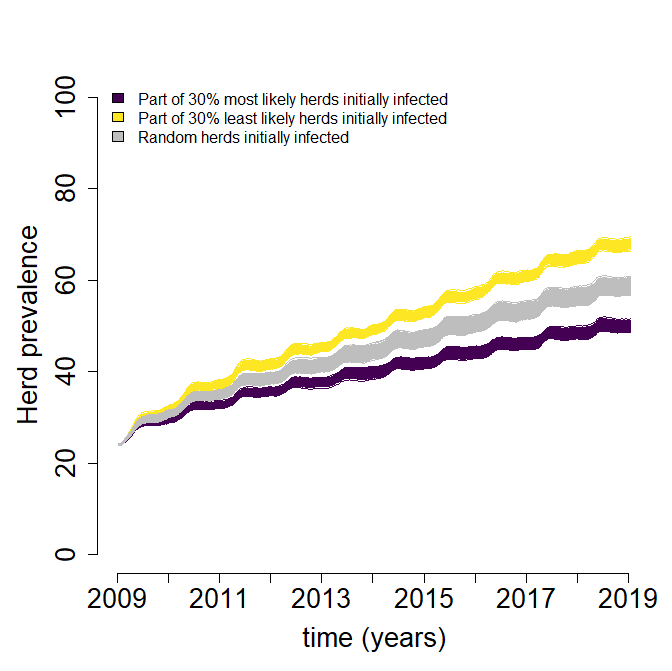


**Figure S5.3. Herd prevalence over time for all replicates.** At the start 25% of the herds were seeded with infection; these herds belonged to the 30% most likely herds to be infected (purple), 30% least likely herds to be infected (yellow), or were randomly selected from the total number of herds (grey).


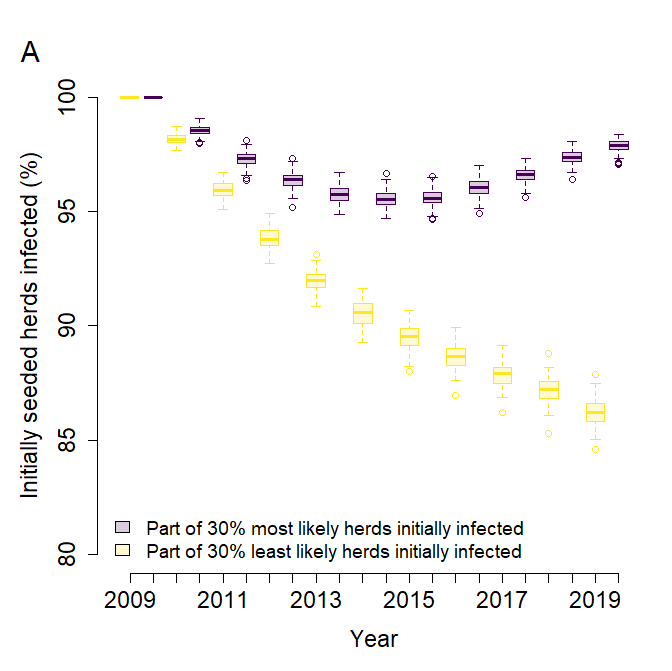

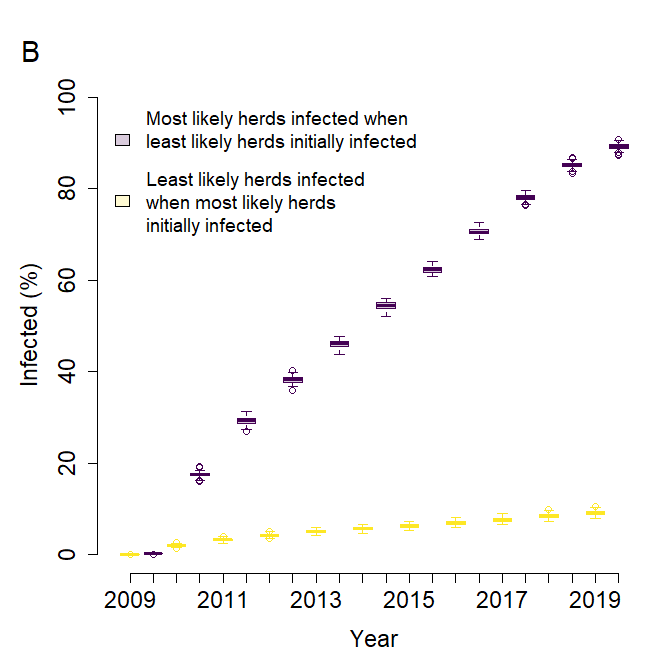


**Figure S5.4 Percentage of most likely (purple) and least likely (yellow) herds infected over time.** A: Percentage of the initially infected herds infected over time when the most likely herds or least likely herds were initially infected. B: Percentage of least likely herds infected over time when the most likely herds were initially infected (yellow) and the percentage of most likely herds infected over time when the least likely herds were initially infected (purple).
