# Supplementary material for: Modelling transmission of Mycobacterium avium subspecies paratuberculosis between Irish dairy cattle herds
Source: Vet Res. 2022 Jun 22;53:45. doi: 10.1186/s13567-022-01066-5 (PMC9215035; doi:10.1186/s13567-022-01066-5)
Supplement: Supplementary file 6 — Additional file 6. Probability distribution from which the initial within-herd prevalence was sampled. [file 13567_2022_1066_MOESM6_ESM.docx]

**Additional file 6**


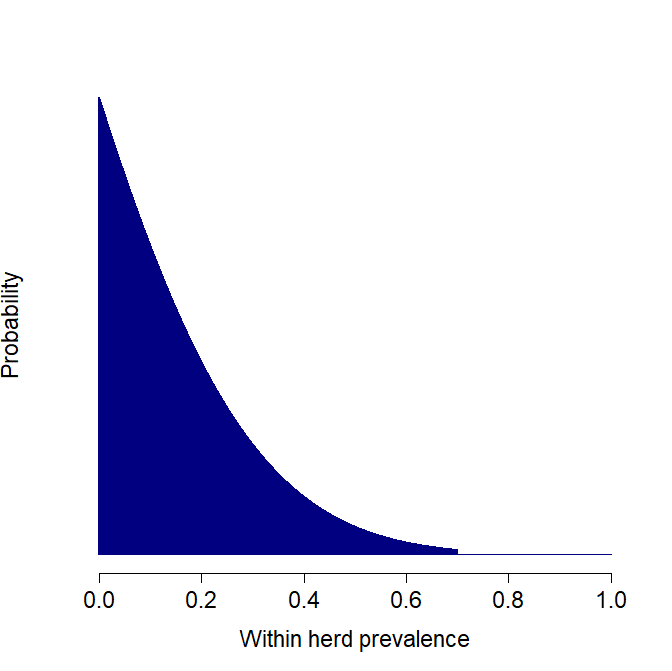


**Figure S6.1. Probability distribution from which the initial within-herd prevalence was sampled.**
